# Counterfactual Diffusion Models Provide Interpretable Explanations of Artificial Intelligence Models in Pathology

**DOI:** 10.1158/0008-5472.CAN-25-5645

**Published:** 2026-06-17

**Authors:** Laura Žigutytė, Tim Lenz, Tianyu Han, Nic G. Reitsam, Sebastian Foersch, Katherine J. Hewitt, Moritz Jesinghaus, Zunamys I. Carrero, Michaela Unger, Asier Rabasco Meneghetti, Georg Lurje, Isabella Lurje, Sophia Herda, Justus Pein, Deniz Uluk, Carolin V. Schneider, Alexander T. Pearson, Daniel Truhn, Jakob Nikolas Kather

**Affiliations:** 1 Else Kröner Fresenius Center for Digital Health (EKFZ), Faculty of Medicine and University Hospital Carl Gustav Carus, TUD Dresden University of Technology, Dresden, Germany.; 2 Department of Diagnostic and Interventional Radiology, University Hospital RWTH Aachen, Aachen, Germany.; 3 Department of Radiology, Perelman School of Medicine, University of Pennsylvania, Philadelphia, Pennsylvania.; 4 Pathology, Faculty of Medicine, University of Augsburg, Augsburg, Germany.; 5 Bavarian Cancer Research Center (BZKF), Augsburg, Germany.; 6 Institute of Pathology, University Medical Center of the Johannes Gutenberg University Mainz, Mainz, Germany.; 7 Institute of Pathology, Philipps University of Marburg, Marburg, Germany.; 8 Department of Gastroenterology and Hepatology, Campus Charité Mitte, Campus Virchow Klinikum, Charité – Universitätsmedizin Berlin, Berlin, Germany.; 9 Department of Surgery, Charité – Universitätsmedizin Berlin, Berlin, Germany.; 10 Division of Transplantation, Department of General Surgery, Medical University of Vienna, Vienna, Austria.; 11 Department of Internal Medicine III, Gastroenterology, Metabolic Diseases and Intensive Care, University Hospital RWTH Aachen, Aachen, Germany.; 12 Section of Hematology/Oncology, Department of Medicine, University of Chicago, Chicago, Illinois.; 13 Chan Zuckerberg Biohub Chicago LLC, Chicago, Illinois.; 14 Department of Medicine I, Faculty of Medicine and University Hospital Carl Gustav Carus, TUD Dresden University of Technology, Dresden, Germany.; 15 Medical Oncology, National Center for Tumor Diseases (NCT), University Hospital Heidelberg, Heidelberg, Germany.; 16 Pathology and Data Analytics, Leeds Institute of Medical Research at St James’s, University of Leeds, Leeds, United Kingdom.

## Abstract

Deep learning can extract predictive and prognostic biomarkers from histopathology whole-slide images. However, explainable artificial intelligence approaches widely used in digital pathology, such as attention heatmaps and class activation mapping, provide limited insight into the image features associated with classifier outputs. In this study, we developed Morphing histoPathology Diffusion (MoPaDi), a framework for generating counterfactual explanations for histopathology images that help identify morphologic or stain-related features linked to model predictions. MoPaDi combined diffusion autoencoders with task-specific multiple instance learning classifiers to manipulate images and induce prediction shifts by modifying classifier-associated features. The framework was evaluated on multiple datasets spanning colorectal, breast, liver, and lung cancers, including tasks for tissue type, cancer subtype, and biomarker [microsatellite instability (MSI)] classification. MoPaDi generated perceptually realistic counterfactual histopathology images, enabling pathologists to identify morphologic features associated with changes in model predictions, complementing the conventional inspection of highly attended regions in digital pathology. In the MSI status prediction task, MoPaDi highlighted morphologic features linked to classifier predictions, including mucinous differentiation, altered glandular architecture, and lymphocytic infiltration, consistent with prior literature. Analyses separating stain-related from morphology-related components suggested that in this setting, prediction changes were predominantly associated with morphology-related rather than stain-related alterations. Overall, MoPaDi is a practical framework for counterfactual explanations in computational pathology that supports the evaluation of model-specific decision cues and hypothesis generation.

**Significance:**

MoPaDi is a diffusion-based tool for counterfactual image generation in cancer histopathology that reveals features associated with deep learning classifier predictions and supports transparent auditing of computational models in biomedical research.

## Graphical Abstract

**Figure:**
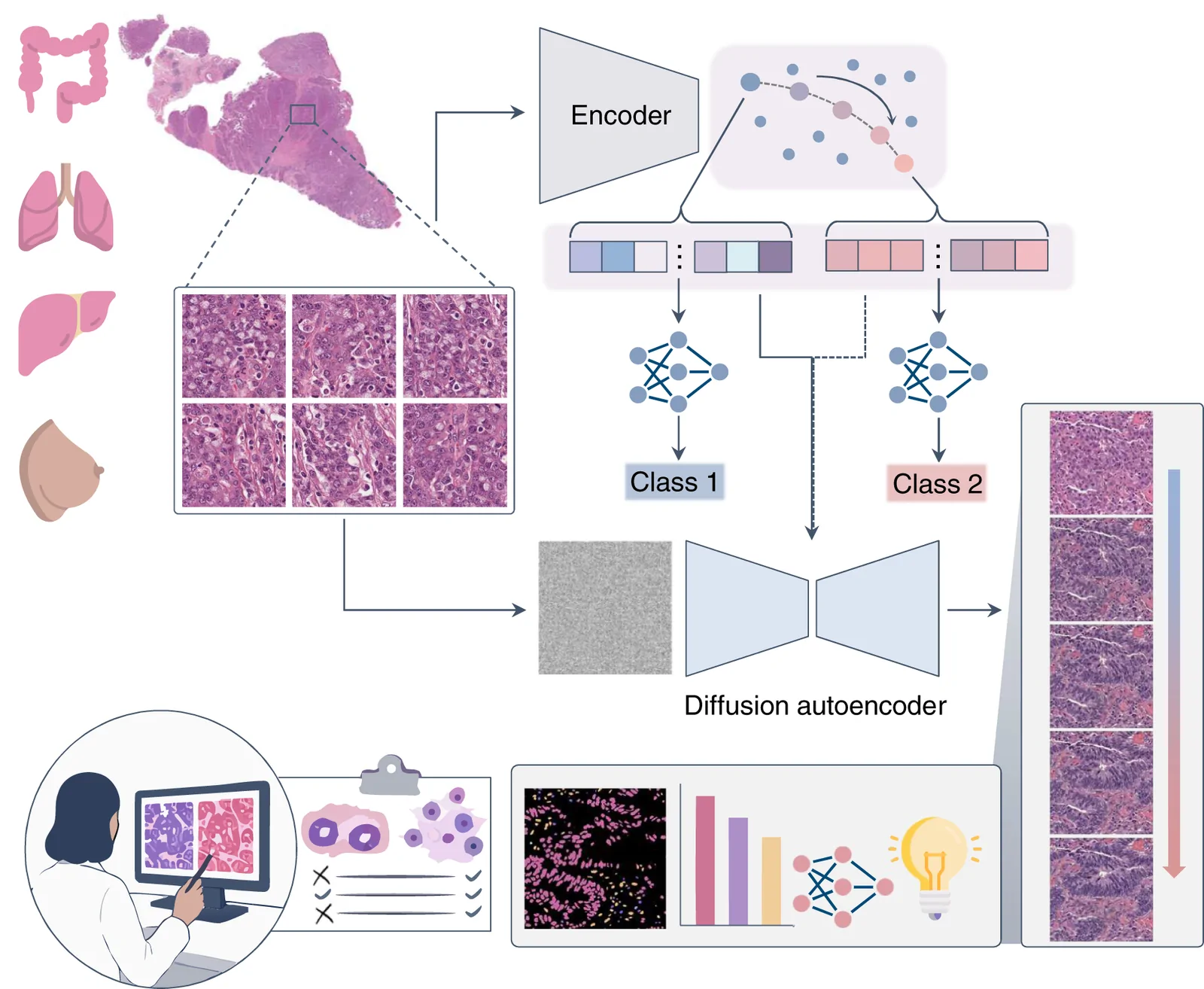

## Introduction

Deep learning (DL) can predict diagnostic and prognostic biomarkers from hematoxylin and eosin (H&E)–stained whole-slide images (WSI) derived from cancer pathology slides (1–4). DL models perform well for many clinically relevant tasks, such as making diagnoses or extracting subtle visual information related to biomarkers (5). However, their so-called black box nature can be problematic, as it is often unclear which features the model relies on to make its predictions, limiting interpretability and trust in the classifier (6). Established explainability methods, such as feature visualization (7) and pixel attribution (e.g., saliency maps, including attention heatmaps; ref. 8), primarily localize influential regions but may provide limited insight into the specific features associated with model predictions (6). Although widely used in biomedical research, these methods are challenging to interpret and may lead to an overestimation of the model’s performance due to confirmation bias (6, 9, 10).

**Figure 1.**
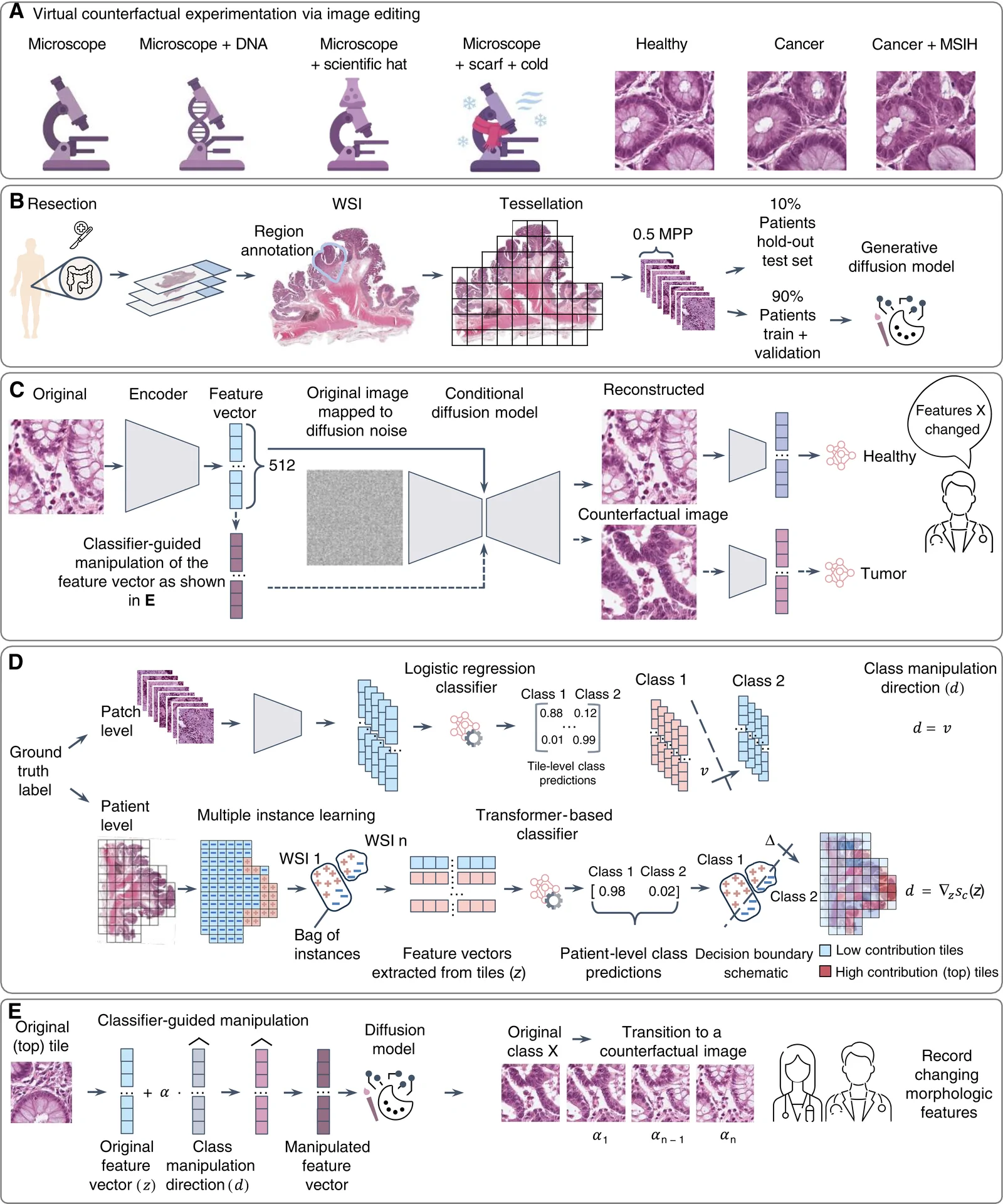
Overview of the experimental setup. **A,** Conceptual illustration showing how semantic modifications to an input can generate counterfactual variants. Analogous to adding attributes to a microscope (left), our method alters key biological attributes in tissue images (right), demonstrating transitions from healthy morphology to cancer and then cancer with MSIH features. “What-if” (counterfactual) edit denotes a model-internal, decision-linked transformation, while not implying biological causality. **B,** Preprocessing steps. Digitized WSIs were resized to 0.5 μm per pixel (MPP), tessellated into 512 × 512 px tiles, and filtered to include only pathologist-annotated tumor regions. **C,** MoPaDi is trained jointly as a feature extractor and a DDPM, conditioned on feature vectors *z*, thereby enabling the reconstruction of the input image. **D,** When a ground-truth label is available at the tile level (NCT-CRC-HE-100K dataset), a logistic regression classifier is trained on tile-level feature vectors extracted with MoPaDi’s encoder to define the decision boundary used for classifier-guided counterfactual generation. Binary classification is shown here for illustrative purposes; the method generalizes to multiclass settings using a one-versus-rest approach. Meanwhile, when a ground-truth label is available at the patient level, an MIL approach is used. MoPaDi learns patient-level predictions from bags of tile features using a transformer-based classifier with attention-based pooling (a more detailed transformer architecture is shown in Supplementary Fig. S2). **E,** For both classifier types, counterfactual images are generated by manipulating the feature vector of a selected tile with a manipulation amplitude α. In tile-level classification, *d* follows the classifier weight vector *v*; in the MIL case, *d* is computed as the gradient of the classifier’s output *s*_*c*_(*z*) for a chosen counterfactual class *c* with respect to the tile’s feature vector *z*. The denormalized manipulated feature vector is then used to condition the diffusion model, yielding a counterfactual image. Pathologists were shown transitions from original to counterfactual images and asked to identify key morphologic changes.

An alternative approach, counterfactual explanations (11, 12), has been explored in other domains, such as natural images, radiology, and dermatology (13–16). Initial generative approaches have also been proposed in computational pathology (16), but diffusion-based counterfactual editing remains underexplored. Counterfactual explanations ask “What if?” questions, such as “How would an image need to change to be classified as class X?” (Fig. 1A). Here, we developed and validated Morphing histoPathology Diffusion (MoPaDi), a framework that generates classifier-guided counterfactuals for histopathology images. Generated images visualize morphologic or style-related changes, such as staining differences, to which the DL classifier is sensitive during prediction. The objective is to make the decision-making of “black-box” artificial intelligence (AI) models more transparent by enabling direct auditing of the visual features associated with their predictions.

Previous studies in other image analysis domains, such as natural images or radiology, have shown that counterfactual explanations can help uncover biases and confounding variables (14, 17) and provide explanations for model predictions. However, these efforts have largely been limited to radiology data or outdated generative AI models such as generative adversarial networks (GAN; refs. 14, 18, 19). Although GAN-based methods have been explored previously (15, 20–25), they are less stable during training and produce lower-quality samples. Therefore, we propose utilizing state-of-the-art diffusion models to generate counterfactual explanations for digital pathology. Diffusion models have surpassed GANs in both synthetic image quality and training stability (26). They work by gradually adding noise to images and learning the reverse process (27). A key strength of diffusion models, due to their iterative nature, is their ability to modify real images while preserving details unrelated to the specific manipulation, thereby allowing fine-grained control over which features are transformed (28, 29). Furthermore, most computational pathology problems require a weakly supervised approach because labels are typically available only for WSIs comprising many tiles (Fig. 1B; ref. 30). Therefore, we extended the counterfactual image generation to the weakly supervised setting by integrating multiple instance learning (MIL; ref. 31) into the framework. We trained attention-based classifiers on patient-level labels and then generated counterfactuals for the top-contributing tiles. MoPaDi combines diffusion autoencoders (29) with MIL to generate high-quality counterfactual images of histopathology tiles, preserving original image details and enabling pathologists to comprehensively evaluate the morphologies captured by the classifier (Fig. 1C–E). To the best of our knowledge, diffusion-based counterfactual image generation has not been previously combined with MIL for weakly supervised histopathology tasks.

The aim of MoPaDi is to reveal which morphologic features in histopathology images are associated with DL classifiers’ predictions by creating counterfactual explanations. Throughout this study, counterfactuals are interpreted as classifier-faithful explanations rather than as direct inferences about biology, and their biological relevance is assessed through orthogonal validation. We first show that the model learns histology-relevant morphologic features by generating tissue-type targeted edits. We then evaluate MoPaDi for its ability to expose discriminative features of a microsatellite instability (MSI) status classifier, a colorectal cancer biomarker for which predictability from H&E-stained WSIs has been widely demonstrated (3, 32). We also show how counterfactual explanations can be used to disentangle model predictions into stain-style and morphology-related components and quantitatively demonstrate that class shifts for MSI predictions are predominantly morphology-driven. Furthermore, we validate MoPaDi across several diagnostically relevant classification tasks in hepatobiliary, lung, and breast cancers. Our framework, validated across multiple datasets and tasks, reveals morphologic features associated with classifier predictions through targeted manipulation of histopathologic images, thereby enabling interpretation of the morphologic patterns to which the DL model is sensitive.

## Materials and Methods

### Experimental design

We designed a multistage evaluation to (i) learn a faithful, decodable latent representation of histopathology images; (ii) train task-specific classifiers at the tile and patient level; and (iii) generate and validate classifier-guided counterfactual images that reveal decision-relevant morphology (Fig. 1).

First, MoPaDi diffusion autoencoders were trained on multiple datasets to enable high-fidelity reconstructions and controllable editing: NCT-CRC-HE-100K; The Cancer Genome Atlas (TCGA) Research Network colorectal cancer cohort (TCGA-CRC); breast cancer cohort (TCGA-BRCA); and a TCGA pan-cancer collection of histopathology tiles. Reconstruction fidelity and generative realism were quantified on test sets using standard quantitative metrics. We then established two classification approaches using features extracted with MoPaDi’s encoder (Fig. 1D): a tile-level logistic regression (one vs. rest) for multiclass tissue typing in colorectal cancer and a weakly supervised, transformer-based MIL model for slide/patient-level tasks.

We then generated counterfactual images to reveal which morphologic changes the classifier is sensitive to by producing visual, model-consistent feature shifts sufficient to alter predictions. We validated effectiveness by summarizing the fraction of tiles in which the predicted class flipped to the target class, confirming trends with independent classifiers, disentangling staining (style) from structure (morphology), and quantifying cellular and structural changes along counterfactual transitions. To assess biological plausibility and perceptual realism, two expert studies were conducted: (i) pathologists annotated original to counterfactual transitions for emerging/disappearing histologic features and (ii) a blinded real-versus-synthetic study measured perceptual realism.

Together, these analyses assessed (i) diffusion autoencoding fidelity for histopathology, (ii) classifier performance, (iii) counterfactual effectiveness, (iv) expert interpretability and perceptual realism, (v) independence from stain/style, and (vi) quantitative cellular correlates—across several cancer types and tasks.

### Datasets and preprocessing

This study explored two colorectal cancer datasets, starting with the publicly available NCT-CRC-HE-100K dataset (1). This dataset comprises 100,000 H&E-stained histopathologic images from 86 formalin-fixed, paraffin-embedded (FFPE) samples representing both human colorectal cancer and normal tissues. The dataset encompasses nine distinct tissue classes: adipose, background, debris, lymphocytes, mucus, smooth muscle, normal colon mucosa, cancer-associated stroma, and colorectal adenocarcinoma epithelium (Supplementary Fig. S1A). All classes were used to train the diffusion autoencoder. Each image had a resolution of 224 × 224 pixels at 0.5 μm per pixel and was color-normalized using the Macenko method (33). The trained model was evaluated on 7,180 tiles from the CRC-VAL-HE-7K dataset, comprising 50 WSIs, which was published alongside NCT-CRC-HE-100K and serves as a nonoverlapping dataset for testing purposes. Further analysis was performed on a second colorectal cancer dataset from TCGA, comprising 625 WSIs obtained from FFPE samples. This cohort was randomly divided at the patient level, allocating 90% for training and 10% for independent testing before any preprocessing. The held-out test set was used not only to evaluate classifier performance but also to assess the quality of image reconstruction, generation, and manipulation. For the TCGA-CRC dataset, in which raw WSIs were processed directly, the slides were tessellated into 512 × 512-pixel tiles at a spatial resolution of 0.5 μm per pixel. WSIs without spatial resolution metadata (*N* = 26) or tumor annotations (*N* = 8) were excluded, resulting in a set of 588 WSIs from 582 patients with colorectal cancer. Patient characteristics and counts per split are provided in Supplementary Table S1. Tiles were derived from tissue regions determined by Canny edge detection. Tiles derived from nontumorous regions were filtered out, retaining only those originating from tumor regions. This selection process was guided by coarse tumor area annotations prepared by pathology trainees and verified by an expert pathologist. Tiles that overlapped with annotated tumor regions by at least 60% were included. No color normalization was performed (except for the NCT-CRC-HE-100K dataset, which was prenormalized) to facilitate the investigation of staining’s influence on classifiers. To determine the threshold between MSI-low (MSIL) and MSI-high (MSIH), we fitted a two-component Gaussian mixture model to the log-transformed distribution of total MSI events [obtained from Cortes-Ciriano and colleagues (34)] across TCGA-CRC samples. We defined the cutoff at the intersection of the two components, with microsatellite stable cases assigned to 0 MSI events, MSIL defined as values below the cutoff, and MSIH as values at or above the cutoff.

Further investigation was conducted on the TCGA-BRCA cohort (1,115 WSIs from 1,046 patients) to investigate counterfactual explanations for breast cancer. This dataset was preprocessed using the same pipeline applied to TCGA-CRC. Finally, we used another publicly available dataset, hereafter referred to as the TCGA Pan-cancer dataset, in which varying-resolution tiles (256 × 256 pixels at 0.5–1 μm per pixel) were extracted from tumor regions of WSIs across 32 cancer types (35). Patient counts in test and train splits are provided in Supplementary Table S2.

To evaluate MoPaDi’s generalizability, we applied the trained classifiers to three external cohorts from the Clinical Proteomic Tumor Analysis Consortium (CPTAC; ref. 36). CPTAC colon adenocarcinoma (CPTAC-COAD) comprised 105 patients (221 WSIs) with MSI status annotations, of which 24 were MSIH. The breast cancer CPTAC cohort comprised 100 patients (323 WSIs) with histologic subtype annotations. The CPTAC lung adenocarcinoma and lung squamous cell carcinoma (LUSC) cohorts comprised 1,534 WSIs from 333 patients (224 and 109, respectively). All CPTAC cohorts consisted of fresh-frozen tissue, representing a tissue-processing domain shift relative to the FFPE samples in the training data. Because no suitable public external cohort including both hepatocellular carcinoma and cholangiocarcinoma was available, we evaluated the liver cancer type classifier on an independent external cohort from Charité – Universitätsmedizin Berlin, comprising H&E-stained slides from patients with liver cancer who underwent resection between 2010 and 2020 (496 WSIs from 496 patients). The slides were digitized at the Else Kröner Fresenius Center for Digital Health using a Leica Aperio AT2 scanner at 40× magnification. Cohort characteristics are provided in Supplementary Table S3. Features were extracted from all validation cohorts without stain normalization, consistent with preprocessing for autoencoder training.

This study was carried out in accordance with the Declaration of Helsinki. We retrospectively analyzed publicly available, deidentified data from TCGA, CPTAC, and Zenodo (histology images from uniform tumor regions in TCGA WSIs and 100,000 histologic images of human colorectal cancer and healthy tissue). These datasets were generated under the ethics approvals of their originating institutions; their secondary use in this study required no additional ethics approval or informed consent. The overall study was approved by the Ethics Commission of the Medical Faculty of the Technical University of Dresden (BO-EK-444102022). The use of liver tumor specimens and pseudonymized clinical data from the Charité cohort was approved by the local ethics committee of the Charité – Universitätsmedizin Berlin (EA1/111/21); the requirement for informed consent was waived by the ethics committee owing to the retrospective analysis of pseudonymized data collected as part of routine clinical care.

### Diffusion autoencoders

MoPaDi’s architecture builds on the official diffusion autoencoder implementation by Preechakul and colleagues (29). It consists of a denoising diffusion probabilistic model (DDPM), conditioned on a feature vector obtained with a learnable convolutional neural network encoder. We refer to this encoder as the feature extractor, which learns to extract meaningful high-level information (feature vectors *z*) from input images (Fig. 1C). The encoder and conditional denoising diffusion model are trained jointly by optimizing a simplified noise prediction loss:

$$L_{\text{simple}}\left(\theta ,\psi \right)=\overset{T}{\underset{t=1}{\sum }}E_{x_{0},\epsilon }\left[{\left|\left|\epsilon_{\theta }\left(x_{t},t,z_{\psi }\left(x_{0}\right)\right)-\epsilon \right|\right|}_{2}^{2}\right]$$ (A)

where *θ* is the denoiser, *ψ* is the encoder, $\epsilon_{\theta }$ is the predicted noise at diffusion time step *t*, *x*_*t*_ is the noisy image at that step, *z*_*ψ*_(*x*_*0*_) is the feature vector from the original image *x*_*0*_, and ϵ*∼ 𝒩*(0,*I*) is the sampled Gaussian noise. Both *θ* and *ψ* are optimized jointly, encouraging the encoder to learn semantically meaningful features while enabling high-fidelity reconstruction.

To train the autoencoding MoPaDi model on the NCT-CRC-HE-100K dataset, we used eight NVIDIA A100 GPUs, each with 40 GB of memory. For the autoencoding model trained on TCGA-CRC, which was tessellated into 512 × 512 px tiles, we used 4 NVIDIA A100 GPUs, each with 80 GB of memory. The models were trained using the DDPM objective, with denoising diffusion implicit model sampling applied at inference for efficient generation. Models were trained until the Fréchet inception distance (FID) score fell below 20, evaluated on 5,000 images. A global batch size of 24 for 512 × 512 px models and 64 for smaller models was used, divided evenly among the GPUs. Models were optimized with Adam (learning rate of 0.0001 and no weight decay), with gradient clipping and an exponential moving average of weights (decay 0.9999); no learning rate scheduler was used. To evaluate the quality of the latent space learned by the autoencoder and generate novel synthetic images, we additionally trained a latent DDPM on the distribution of feature vectors extracted with MoPaDi’s encoder.

Model development was performed in Python 3.11 using PyTorch 2.7.0. A reproducible *uv* environment with pinned dependencies is provided in the public GitHub repository (https://github.com/KatherLab/mopadi).

### Classifiers and counterfactual image generation

To enable classifier-guided counterfactual image generation, we first establish classification models for tile- and patient-level ground truth labels. For tile-level tasks, such as tissue-type classification, we trained a multiclass logistic regression classifier on 512-dimensional features extracted with pretrained MoPaDi’s encoder (Fig. 1D). Each tile is annotated with a one-hot target vector indicating its true class. Features are normalized to zero mean and unit variance before classification. Although the task is multiclass, we treat it as a one-versus-rest problem by applying binary cross-entropy loss independently to each class. The classifier was trained on 300,000 total samples with a batch size of 32 (9,375 iterations).

For patient-level ground truth labels, such as biomarker status (e.g., MSI), we adopted an MIL approach using transformer-based pooling. A transformer-based classifier processes bags of tile-level features, applying layer normalization, multihead cross-attention, and a self-attention block, followed by a classifier head with two fully connected layers (with dropout and sigmoid linear unit activation; ref. 37), and a final linear output layer (Supplementary Fig. S2). The classifiers were trained using the Adam optimizer, a learning rate of 0.0001, early stopping with a patience of 20 epochs, and cross-entropy loss with inverse class proportion weighting to address class imbalance. Model performance was assessed via fivefold cross-validation. For counterfactual image generation, a final classifier is trained on the full training set over 50 to 200 epochs, depending on cross-validation results.

Both the linear and MIL classifiers guided counterfactual image generation by defining a class-specific direction of manipulation in feature space. In the linear case, the learned weight vector *v* of the logistic regression model defined the direction *d* in feature space that increases the logit for the target class. This direction is used to shift the tile’s feature vector across the decision boundary. In the MIL setting, where the relationship between individual tiles and the label is nonlinear, we computed the gradient of the classifier’s output for a chosen counterfactual class *c* with respect to the selected tile’s normalized feature vector *z* and used this gradient as the direction of manipulation:

$$d\;=\;\nabla_{z}s_{c}\left(z\right)$$ (B)

where *s*_*c*_(*z*) denotes the raw logit that the classifier assigns to a class c given a feature vector *z*.

After modifying the original feature vector z by adding *d* [scaled by the manipulation amplitude α and adjusted for feature dimensionality, i.e., 512 (Eq. C)], the updated feature vector *ẑ* is denormalized and passed to the conditional diffusion decoder (*D*), along with the original image inverted to noise (*x*_*T*_) to generate the counterfactual image *◯* (Fig. 1E; Eq. D).

$$ẑ\;=\;z\;+\;\alpha \;\cdot \;\sqrt{512}\cdot \;d$$ (C)

$$\hat{x}=\;D\left(x_{T},\;ẑ\right)$$ (D)

We apply counterfactual manipulation by progressively increasing *α* along the target-class gradient direction in latent space. For each dataset, we used a fixed set of manipulation amplitudes [e.g., α ∈ (0.2, 0.4, 0.6, 0.8, 1) for the linear approach and α ∈ (0.02, 0.04, 0.06, 0.08) for the MIL approach]. Even if some manipulated tiles do not cross the classifier’s decision boundary at the largest *α*, we applied the same *α* values uniformly across the cohort to ensure comparability. We defined the manipulation amplitudes by increasing *α* in preliminary analyses until the majority of counterfactual images were classified as the target class. The predictions reflect what the classifier would predict if it were given only that tile. For the MIL approach, counterfactual images can be generated for any number of selected tiles. In this study, we generated counterfactual images for the top five most contributing tiles per WSI, as a compromise between interpretability, coverage of high-evidence regions, and computational burden. Because each tile is manipulated independently, this choice determines the number of regions analyzed rather than the counterfactual generation process for an individual tile. For fixed model weights, input image, manipulation amplitude, and denoising diffusion implicit model sampling parameters, counterfactual generation is deterministic because the feature vector and noise representation are inferred from the input image and kept fixed during deterministic sampling.

### Evaluation of classifiers and reconstructions

We assessed the autoencoder’s reconstruction quality on a randomly selected set of 1,000 images per dataset using the mean squared error (MSE) between the original and reconstructed pixel intensities and the multiscale structural similarity index measure (MS-SSIM; ref. 38). Perceptual similarity was additionally quantified using the learned perceptual image patch similarity metric with an AlexNet backbone, and semantic feature preservation was assessed by computing the cosine similarity between UNI2 (39) feature embeddings of original and reconstructed tile pairs. In addition, the structural similarity index (40) was used to visualize regions of similarity and dissimilarity between the original and reconstructed images after grayscale conversion to facilitate interpretation. The ability of the latent space to retain semantically meaningful information was further evaluated by training an additional model on feature vectors extracted with MoPaDi’s encoder and sampling 10,000 synthetic images. To compare the distributions of real and generated images, we computed the FID score using the clean-fid library (41), with lower scores indicating greater similarity between real and synthetic image distributions. To complement FID with pathology-relevant metrics, we also computed the Fréchet distance using feature embeddings extracted with two pathology foundation models, UNI2 (39) and Virchow2 (arXiv 2408.00738), thereby providing a domain-aware assessment of generative image fidelity.

To evaluate classifier performance, we computed the area under the receiver operating characteristic (ROC) curve on the held-out test set. Bootstrapping was employed as a resampling technique to assess robustness and confidence intervals (CI). Resampling was done with replacement 1,000 times. We constructed ROC curves for each resampled set and interpolated between them to obtain a set of smoothed curves. Ninety-five percent CIs were extracted from smoothed curves. The latent space and the direction of manipulation were visualized using t-distributed stochastic neighbor embedding (t-SNE), providing a qualitative representation of extracted 512-dimensional feature vectors in a reduced 2-dimensional format.

### Style–morphology decomposition of prediction changes

To quantify the relative influence of stain style versus tissue morphology on model predictions, we performed a Shapley-based style–morphology decomposition using pairs of original and counterfactual images. For each tile *x* (original) and its counterfactual *x*_*cf*_, we defined the total prediction change for the target class *c* as follows:

$$\Delta f_{total}\;=\;s_{c}\left(E\left(x_{cf}\right)\right)\;-\;s_{c}\left(E\left(x\right)\right)$$ (E)

where *s*_*c*_(·) denotes the raw classifier logit.

To separate this change into style- and morphology-related components, we created two hybrid images:

- *x*_*style*_—preserving the morphology of the original tile while adopting the stain style of the counterfactual
- *x*_*morph*_—preserving the stain style of the original while adopting the morphology of the counterfactual

Stain style transfer was performed using the Vahadane method (42). Predictions on these hybrids were used to compute first-order effects of style and morphology. Following a Shapley-style additive decomposition, the total change was partitioned into

$$\Delta f\;=\;\varphi_{style}\;+\;\varphi_{morph}$$ (F)

where *φ*_*style*_ and *φ*_*morph*_ represent the respective contributions of stain style and tissue morphology to the overall logit change, including half of their shared interaction term so that the sum equals the total change.

Predictions were obtained from the same MIL classifier used in the main experiments. The decomposition was applied per tile, and per-patient statistics were computed as the median of tile-level values. Because stain transfer methods such as Vahadane may not achieve perfect separation of stain and morphology, the resulting decomposition should be interpreted as an approximate attribution of the change in prediction to staining versus morphologic differences, rather than as an exact disentanglement.

Gradient-weighted Class Activation Mappings (Grad-CAM) were generated by backpropagating from the target-class logit through the encoder’s last convolutional layer, computing gradient-weighted activations, and overlaying the resulting heatmap on the input tile.

### User studies

To evaluate whether MoPaDi-based classifiers capture meaningful morphology, we conducted task-specific expert studies that included both structured feature-level surveys and qualitative reviews of representative examples. For structured feature-level evaluation of class-specific counterfactual transitions, three board-certified pathologists independently reviewed original and counterfactual image pairs. For the normal colon mucosa versus tumor task, an additional pathology trainee also participated. For each transition, 4 to 5 images were shown in sequence (original → intermediate steps → counterfactual), and raters were asked to indicate morphologic features present in the leftmost (original) and rightmost (counterfactual) images. A predefined checklist of task-relevant histopathologic features was provided, and raters were instructed to select all applicable features for both original and counterfactual images. The survey consisted of 52 image transitions for the MSI task (13 patients per class × 2 top-contributing tiles per patient), 30 transitions for the normal-versus-tumor task, and 32 transitions for the lung cancer types task, yielding two feature annotations per transition (original and counterfactual).

For the breast and liver classifiers, evaluation was performed as a qualitative review rather than a predefined feature survey. Two raters reviewed 16 examples per classifier (8 per class). Each example consisted of two top-predictive tiles from the same correctly classified patient, together with their counterfactual transitions, and the raters summarized the morphologic changes associated with the class shift.

Finally, to further assess the perceptual realism of the generated counterfactual images, four raters (three board-certified pathologists and one pathology trainee) reviewed 300 images (150 real and 150 synthetic, randomly selected) in a blinded setting. For each task, 30 real tiles (15 per class) were randomly selected from the test set, and corresponding counterfactuals were generated by transitioning each tile to the opposite class. The raters were presented with a mixed set of tiles and asked to classify each one as either real or synthetic. The proportion of correctly identified images served as a measure of the diffusion model’s ability to generate visually convincing histopathology images.

### Independent classifiers for counterfactual evaluation

To assess whether MoPaDi-generated counterfactuals produce morphologically consistent class shifts, we employed independent classifiers trained on histopathology features extracted from large-scale foundation models. These classifiers were based on the solid tumor associative modeling in pathology (STAMP) framework (43), which integrates pretrained vision encoders with MIL for patient-level prediction. Using the same TCGA-CRC training split, we trained three classifiers using features extracted with UNI2 (39), Virchow2 (arXiv 2408.00738), and CONCH (44) models. For each feature extractor, tile-level features were aggregated at the patient level using the STAMP MIL transformer. All classifiers were trained to distinguish MSIH from the absence of this biomarker (hereafter referred to as non-MSIH) using categorical cross-entropy loss. Each classifier was applied to both the original and counterfactual images. Predictions were obtained independently for every manipulation amplitude, yielding per-tile output for both classes. We quantified classifier agreement with the intended direction of manipulation by analyzing classifier output trajectories as manipulation amplitudes increased. Specifically, for each tile and feature extractor, we measured the change in predicted target-class probability [Δ*P*(*target*)] as a function of amplitude. Aggregated trajectories and mean Δ*P*(*target*) values were visualized to assess whether counterfactuals consistently increased the likelihood of the target phenotype across feature extractors.

### Quantitative assessment of cellular and morphologic changes

Pretrained nuclei segmentation and cell type classification models from DeepCMorph (45) were applied to both original and counterfactual images to quantify cell type proportions and morphologic characteristics. DeepCMorph models were pretrained on H&E tiles at varying resolutions (224 × 224 px) from diverse datasets to classify nuclei from six cell types: epithelial, connective tissue, lymphocytes, plasma cells, neutrophils, and eosinophils. Although originally optimized for cancer and tissue-type classification, the models capture biologically meaningful cellular features that enable accurate segmentation-based quantification.

For epithelial cells, instance segmentation masks were derived by performing watershed-based splitting of touching objects within a semantic segmentation mask, followed by additional binary erosion to split remaining touching nuclei, connected-component labeling, and binary dilation to restore the original nucleus size. Nuclei smaller than 30 pixels were discarded as artifacts. Connective tissue nuclei, which are more elongated and spatially separated, were segmented directly from the predicted masks without further postprocessing.

From the resulting instance masks, we extracted morphologic and intensity-based measurements, that is, area, mean intensity, and eccentricity, using the *scikit-image* library. To capture spatial tissue organization, we computed the entropy of cellular spatial distributions by binning cell centroids into a 2-dimensional histogram, normalizing it to a probability distribution, and calculating Shannon entropy, which was then normalized by the maximum possible entropy given the histogram resolution. We also quantified cell counts per type and computed cell type fractions (relative proportions).

For each patient, changes were assessed by comparing the medians of these measurements between the counterfactual and original images. The direction of change was signed such that positive values indicate a shift toward MSIH morphology. Cohort-level summaries were computed as median signed deltas across patients, with interquartile ranges (IQR) used to estimate variability. Statistical significance was evaluated using two-sided Wilcoxon signed-rank tests.

To quantitatively compare changes in cell-type proportions between original and counterfactual images of the opposite class (i.e., images manipulated to resemble the class being compared), we performed statistical testing at the patient level for the MSI classifier and at the tile level for the remaining tasks. For each patient and cell type, the mean proportion across analyzed tiles was computed separately for the original and manipulated images. Differences between original and counterfactual distributions were assessed using the two-sided Mann–Whitney U test as the data were non-normally distributed and unpaired across patients. *P* values were adjusted for multiple comparisons across cell types using the Bonferroni correction (familywise α = 0.05). Effect sizes were quantified as the Hodges–Lehmann estimator of the median shift (in percentage points) with a 95% percentile-bootstrap CI and complemented by Cliff’s delta (δ), interpreted using conventional thresholds (|δ| < 0.147 = negligible; <0.33 = small; <0.474 = medium; and ≥0.474 = large).

## Results

### Diffusion autoencoders faithfully represent and reconstruct histopathology images

MoPaDi is a diffusion-based model that preserves global tissue architecture while capturing fine cellular detail in its latent representation, enabling accurate reconstruction of histopathology images. To assess the fidelity of these latent representations, we first quantified reconstruction performance across multiple settings by training four independent models on the following datasets: TCGA-CRC, TCGA-BRCA, TCGA pan-cancer, and the NCT-CRC-HE-100K dataset. We observed consistently high image reconstruction performance, reflected in low MSE and high MS-SSIM values across datasets (Table 1). The qualitative review further demonstrated near-identical reconstructions, with only subtle artifacts occasionally accentuated during the reconstruction process (Supplementary Fig. S3).

**Table 1.** Evaluation of diffusion autoencoders trained on different datasets.

Model	MS-SSIM ↑	MSE ↓	LPIPS ↓	Cos sim (UNI2) ↑	FID_gen_ ↓	FD_gen_ V2 ↓	FD_gen_ UNI2 ↓
CRC-VAL-HE-7K	0.968 ± 0.036	(0.4 ± 0.5) × 10^−3^	0.060	0.882 ± 0.093	36.95	511.44	131.03
TCGA pan-cancer	0.992 ± 0.001	(0.8 ± 0.2) × 10^−3^	0.051	0.947 ± 0.033	4.71	86.53	27
TCGA-CRC	0.967 ± 0.060	(8.1 ± 14) × 10^−3^	0.170	0.837 ± 0.133	16.85	200.85	47.37
TCGA-BRCA	0.966 ± 0.062	(4.5 ± 11.3) ×10^−3^	0.171	0.805 ± 0.176	17.85	214.10	48.46

NOTE: Reconstruction quality was assessed on 1,000 randomly sampled tiles using MS-SSIM, MSE, learned perceptual image patch similarity (LPIPS), and cosine similarity of UNI2 feature embeddings between original and reconstructed tiles. The fidelity of the learned latent space was further evaluated by generating 10,000 synthetic images and computing the FID score between generated and held-out test set images, as well as Fréchet distances (FD) in UNI2 and Virchow2 (V2) feature spaces.

We further assessed the encoder’s ability to capture meaningful features by training a diffusion model on feature vectors extracted with MoPaDi’s dataset-specific encoders, enabling the sampling of synthetic feature vectors for downstream image generation. These models produced high-quality synthetic images, as indicated by low FID between a held-out set of original images and 10,000 generated synthetic images (Supplementary Fig. S4) for most datasets (Table 1). The highest FID score of 36.95 was obtained by the model trained on NCT-CRC-HE-100K when evaluated on the CRC-VAL-HE-7K dataset, reflecting the greater histologic heterogeneity of this dataset, which covers nine distinct tissue classes rather than tumor regions alone. The notably low FID of 4.71 for the pan-cancer model likely reflects a low-diversity reference set. The overall pattern was consistent with complementary Fréchet distances computed using Virchow2 and UNI2 features. Together, these results demonstrate that MoPaDi effectively captures key image features and reconstructs histopathology tiles, providing a robust foundation for downstream counterfactual image generation.

### Tissue type transformations via latent editing exhibit meaningful patterns

After validating MoPaDi’s reconstruction performance and latent structure, we next examined whether the model can manipulate these representations to generate plausible counterfactual images across tissue types. For this, we first trained a diffusion autoencoder alongside a feature extractor on the NCT-CRC-HE-100K dataset, representing nine tissue classes in colorectal cancer (Supplementary Fig. S1A). Subsequently, we trained a logistic regression classifier. For generating counterfactual explanations, we focused on the most clinically relevant pair: normal colon mucosa and dysplastic/tumor epithelium. Evaluated on the independent CRC-VAL-HE-7K dataset, the classifier reached an area under the curve (AUC) of 0.91 ± 0.01 for normal colon mucosa and 0.98 ± 0.01 for tumor epithelium (95% CI). AUC values for the remaining classes ranged from 0.79 to 1 (Supplementary Fig. S1B), and counterfactual images can also be generated for these classes (representative examples are shown in Supplementary Fig. S5).

**Figure 2.**
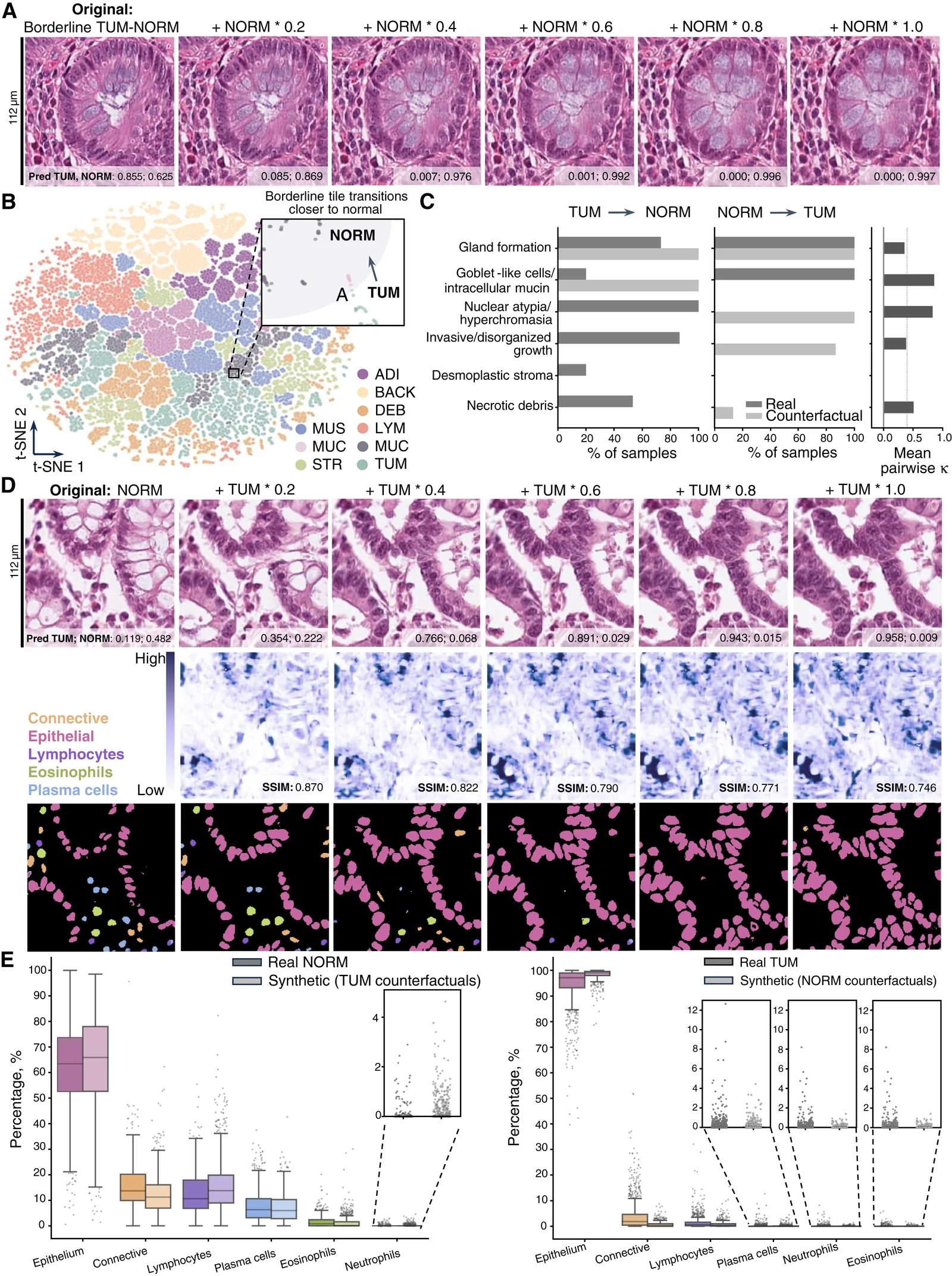
Results for counterfactual image generation for the CRC-VAL-HE-7K dataset. **A,** Tissue-type classifier-guided counterfactuals of a dysplastic tile, generated by shifting its feature vector toward the predicted healthy colon mucosa class. Changes reflect what the model requires to flip the prediction, with increasing manipulation amplitude shown above each image. **B,** The t-SNE projection of the feature extractor’s latent space (training set), colored by class: adipose (ADI), background (BACK), debris (DEB), lymphocytes (LYM), mucus (MUC), smooth muscle (MUS), healthy colon mucosa (NORM), cancer-associated stroma (STR), colorectal adenocarcinoma epithelium/dysplastic tissue (TUM). The features of dysplastic tile in **A**, initially near the boundary between TUM (aqua) and NORM (gray), shift toward the healthy cluster (pink points in the zoomed-in plot; gray area—arbitrary healthy mucosa zone) as manipulation amplitude increases. **C,** Morphologic feature prevalence in original and counterfactual image pairs. Horizontal bars show the percentage of image pairs in which at least one of four raters (three board-certified pathologists and one pathology trainee) identified each morphologic feature as present in the original (dark gray) or counterfactual (light gray) image. Right, Mean pairwise inter-rater agreement (Cohen κ) per feature, computed across all pairs and both directions combined. **D,** Counterfactuals of a healthy mucosa tile shifted toward dysplastic epithelium. Below, Pixel-level difference maps (darker regions indicate greater changes), showing the most change in gland regions, and SSIM values, which quantify the similarity between the original and manipulated tiles. Bottom, Segmented and classified nuclei: pink (epithelial), orange (connective tissue), blue (plasma), deep purple (lymphocytes), aqua (neutrophils), and green (eosinophils). **E,** Tile-level differences in cell type fractions between (left) real NORM tiles (*n* = 741) and synthetic (counterfactual images generated from TUM tiles, *n* = 1,233; full statistical details are provided in Supplementary Table S4). Right, Tile-level differences in cell-type fractions between real TUM tiles (*n* = 1,233) and synthetic (counterfactual images generated from NORM tiles, *n* = 741).

We used the classifier to shift the latent features toward a selected target class. The manipulated features then conditioned the diffusion-based decoder, enabling us to generate counterfactual images for tiles from the CRC-VAL-HE-7K dataset at increasingly larger manipulation amplitudes (α; representative example of generated transitions in Fig. 2A and additional examples in Supplementary Fig. S6). When visualized in 2-dimensional space, features extracted with MoPaDi’s encoder from the training dataset clustered by tissue type, suggesting that the model captured class-specific representations (Fig. 2B). Manipulated features also shifted closer to the opposite class (Fig. 2B zoomed-in plot), particularly when features are near the class boundary. To quantify the effectiveness of counterfactual generation, we computed the percentage of manipulated tiles predicted to belong to the opposite class across increasing amplitudes (Supplementary Fig. S1C). We observed that transitions from normal colon mucosa to tumor epithelium generally required lower amplitudes to change the classifier prediction, consistent with the lower AUC for the healthy class.

In an evaluation study, four raters assessed which morphologic features changed in counterfactual images (*N* = 30 transitions; 15 per class; Fig. 2C). For malignant epithelium to healthy mucosa transitions, counterfactuals showed the disappearance of nuclear atypia/hyperchromasia, invasive/disorganized growth, and necrotic debris, together with the emergence of goblet-like cells, consistent with a shift toward healthy mucosa morphology. Conversely, transitions from normal mucosa to malignant epithelium showed the opposite pattern, with loss of goblet-like cells, emergence of nuclear atypia, and invasive/disorganized growth, consistent with a shift toward colorectal adenocarcinoma epithelium (Fig. 2D). Taken together, these transitions indicate that MoPaDi captured the distinction between malignant epithelium and healthy mucosa.

We further evaluated the quality of the generated images quantitatively by analyzing the distribution of six cell types in real and synthetic images (i.e., epithelial cells, connective tissue cells, lymphocytes, plasma cells, eosinophils, and neutrophils; Fig. 2E). Cell type composition differences were generally small or negligible (Supplementary Table S4). Across the six cell-type comparisons, all cell types, except plasma cells, showed significant tile-level differences between original healthy colon mucosa tiles (*n* = 741) and tumor tiles manipulated to be classified as healthy class (*n* = 1,233; *P* < 0.05; Mann–Whitney U, Bonferroni-corrected), but all observed effect sizes were negligible to small (Cliff’s delta <0.33; all Hodges–Lehmann median shifts ≤3.1 percentage points). When comparing real tumor epithelium tiles (*n* = 1,233) and their counterparts generated from healthy mucosa tiles (*n* = 741), the analysis revealed small but statistically significant shifts in connective tissue, lymphocytes, plasma cells, and epithelium fractions (*P* < 0.05), with large and medium effect sizes for the connective and epithelial cell fractions, respectively. On average, the epithelial cell proportion was 1.57 percentage points higher in the synthetic images than in the real images (95% CI, 1.34–1.83) and 1.27 percentage points lower for connective tissue cells (CI, 1.07–1.47), suggesting that MoPaDi has captured epithelial cell abundance as a key feature distinguishing malignant epithelium.

Collectively, these findings demonstrate that our model captured and could generate histologic features associated with different tissue types.

### Counterfactuals reveal classifier-learned MSI-associated morphology

Having established that MoPaDi generates biologically meaningful counterfactuals for tissue classification, we next evaluated its ability to predict a molecular biomarker from histopathology and to reveal the features the classifier used to make the prediction. We explored the link between genotype and phenotype by investigating counterfactual explanations for MSI in the TCGA-CRC cohort. A fivefold cross-validation was performed with MIL to predict MSIH versus non-MSIH, achieving a mean AUC of 0.73 ± 0.08 and a mean average precision (AP) of 0.40 ± 0.09, with lower AP reflecting high class imbalance. To assess generalizability, we evaluated the classifier on frozen tissue from the independent CPTAC-COAD cohort (105 patients; 221 WSIs), which achieved an AUC of 0.67, suggesting partial transfer across tissue-processing protocols despite the known domain shift between FFPE and frozen tissue. Given the moderate predictive performance, we treated the MSI model primarily as a case study for auditing classifier behavior.

**Figure 3.**
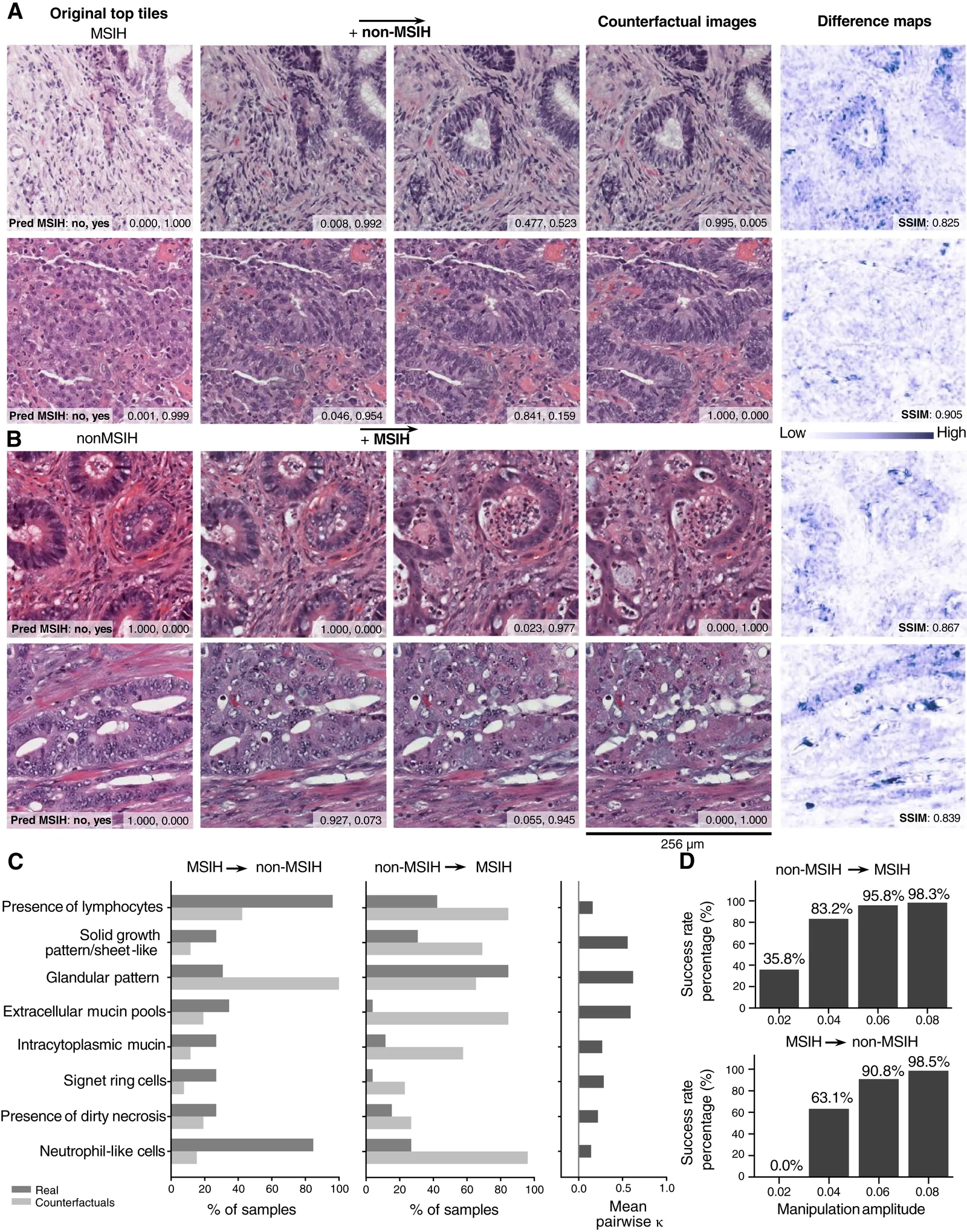
Interpretation of the molecular biomarker (MSI) classifier in colorectal cancer through counterfactual image analysis. **A,** Representative examples of MSIH tiles transitioning to their counterfactual non-MSIH image. Pixel-wise difference images compare the original image with the most manipulated one. **B,** Representative examples of non-MSIH tiles transitioning to their counterfactual MSIH image. Pixel-based difference maps highlight changes in nuclei, stroma, cell density, and tissue architecture. **C,** Morphologic feature prevalence in original and counterfactual image pairs (*N* = 52 transitions; 26 tiles from 13 patients for each class). Horizontal bars show the percentage of image pairs in which at least one of three raters (three board-certified pathologists) identified each morphologic feature as present in the original (dark gray) or counterfactual (light gray) image. Right, Mean pairwise inter-rater agreement (Cohen κ) per feature, computed across all pairs and both directions combined. **D,** Quantitative evaluation of counterfactual image generation effectiveness. Bars show the percentage of successful manipulations (i.e., predicted class equals target class) across different manipulation amplitudes. The scale bar applies to all images within the figure.

We generated counterfactuals for the top five contributing tiles per WSI and examined whether counterfactuals revealed morphologic changes consistent with reported MSI-associated patterns. These changes were subsequently evaluated through an expert user study involving three board-certified pathologists and through quantitative analyses. Counterfactual transitions from MSIH to non-MSIH suggested that evidence for MSIH decreased as glandular architecture became more organized, mucin content decreased, and solid or sheet-like growth patterns were reduced (Fig. 3A; Supplementary Fig. S7). Conversely, counterfactual edits from non-MSIH to MSIH suggested that evidence for MSIH increased as glandular architecture was lost, solid growth patterns became more prominent, and mucinous features increased (Fig. 3B; Supplementary Fig. S8). Pathologist survey results are summarized quantitatively in Fig. 3C. Furthermore, to assess whether MoPaDi could also help investigate more ambiguous regions beyond the strongest slide-level evidence, we additionally generated counterfactuals for borderline tiles with prediction scores close to the classifier decision threshold (Supplementary Fig. S9). These tiles could also be manipulated toward the opposite class and often required smaller amplitudes to flip, consistent with their closer proximity to the classifier decision boundary (Supplementary Fig. S9B). Furthermore, many of the generated images for both classes revealed center-specific batch effects through visible color changes. Quantitative measurements supported these observations, showing significant changes in mean nuclear intensity for both epithelial and connective tissue cells (Supplementary Fig. S10A). These observations motivated us to analyze the relative contributions of style and morphology to model decisions.

We further assessed changes in cell type composition and cell-level morphologic metrics, including nuclear area, solidity, and eccentricity, during counterfactual transitions (Supplementary Fig. S10). Counterfactual edits from MSIH to non-MSIH exhibited a reduction in immune cells and a corresponding increase in epithelial and connective tissue fractions, whereas non-MSIH to MSIH transitions displayed the opposite trend, with a significant increase in immune cells (Supplementary Fig. S10B). These shifts align with the well-established immune-rich microenvironment of MSIH tumors and reinforce that MoPaDi captures biologically meaningful tissue-level features. Notably, when comparing cell type fractions between real images of a given class and counterfactual images manipulated toward that class, no significant differences were detected, suggesting that MoPaDi accurately captured tissue composition and generated class-consistent representations (Supplementary Fig. S10C; statistical details in Supplementary Table S5).

**Figure 4.**
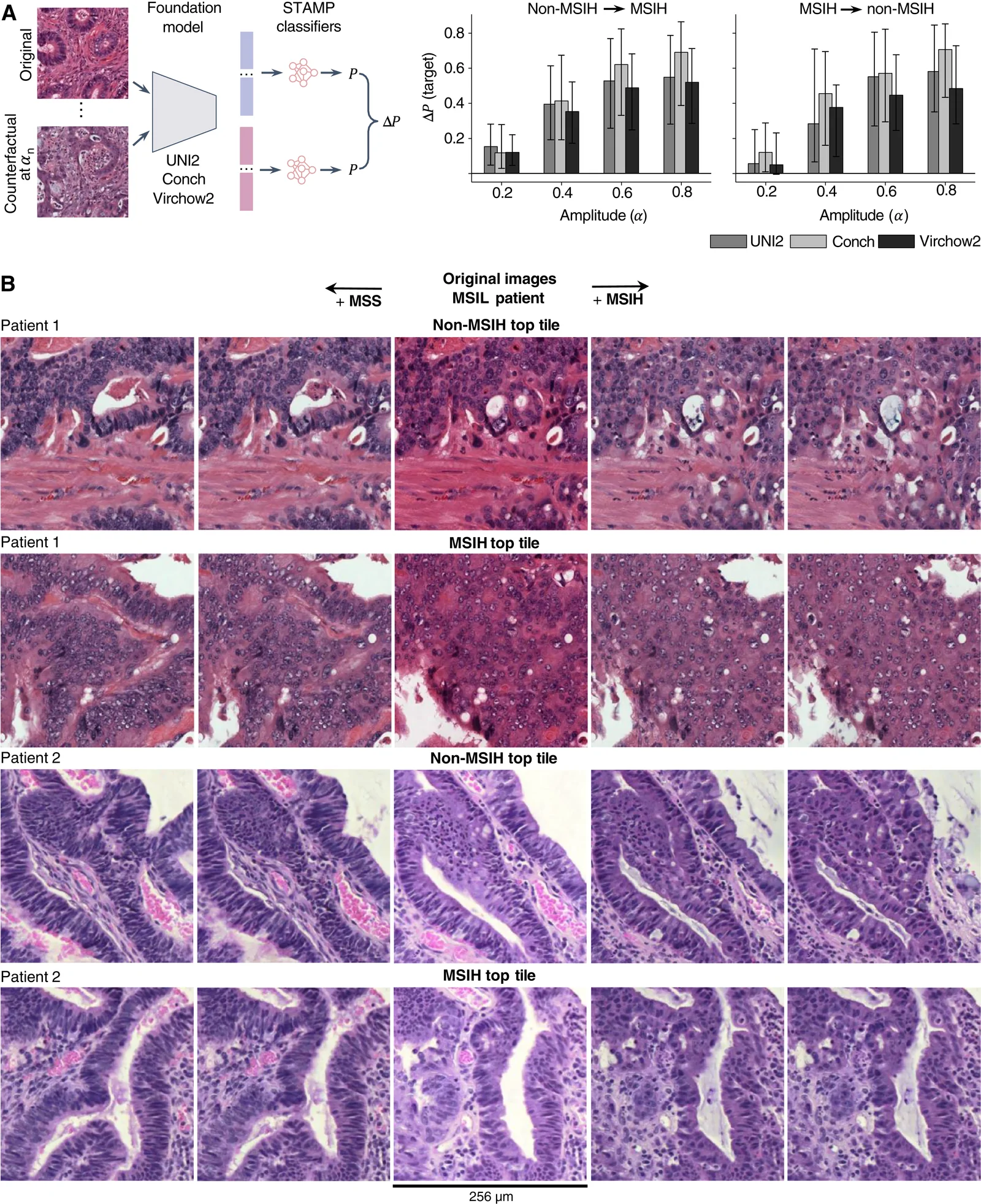
Independent classifier validation and representative bidirectional counterfactual transformations in a multiclass setting. **A,** External classifier responses to counterfactual morphing. Counterfactual images were generated at increasing morphing amplitudes (α) with MoPaDi and then encoded with three foundation models (UNI2, CONCH, and Virchow2). Independent classifiers were trained on the corresponding encoders’ features extracted from all TCGA-CRC tiles and then used to predict the target probability *P*(*target*) on both the original and counterfactual tiles. The resulting change Δ*P*(*target*) reflects how strongly the morphing affected class evidence. Bars show the median Δ*P*(*target*) with percentile-based variability across tiles. **B,** Representative examples of bidirectional counterfactual explanations for MSIL patients. We defined MSIL by fitting a two-component Gaussian mixture model to the log-transformed distribution of total MSI events and using the intersection of the two components as the cutoff separating MSIL from MSIH samples.

Additionally, we assessed both the robustness and effectiveness of our counterfactual generation approach across increasing manipulation amplitudes (α). Most counterfactuals successfully reached the target class even at low α values, demonstrating efficient movement along the model’s decision boundary (Fig. 3D). To assess robustness, we examined whether independent histopathology classifiers agreed with the direction of the counterfactual changes. We applied three state-of-the-art foundation model-based classifiers (UNI2, CONCH, and Virchow2) to both original and counterfactual tiles and quantified changes in MSI prediction confidence as manipulation amplitude increased. Consistent shifts in MSI probability in the intended direction across all classifiers confirmed that counterfactual trajectories are supported by external models (Fig. 4A; Supplementary Fig. S11).

Generated images should not only flip the class but also remain visually convincing. We evaluated perceptual realism through a blinded review by four raters, each assessing 300 tiles as either real or synthetic (Supplementary Fig. S12). Across all datasets, synthetic tiles were misclassified as real in 41.7% of cases, indicating that MoPaDi-generated images were often perceived as realistic by expert observers. All four raters showed higher sensitivity than specificity, consistent with a tendency to classify ambiguous tiles as real rather than synthetic. Together, these findings support the perceptual realism of MoPaDi-generated counterfactual images.

We next evaluated whether our framework can be applied to the more nuanced three-class MSI setting. MSI status is clinically categorized as microsatellite stable, MSIL, or MSIH based on the fraction of tested microsatellite loci in which instability is detected. To qualitatively assess MoPaDi in this multiclass context, we generated counterfactual images for representative MSIL patients, selecting tiles with high model attention toward either the non-MSIH or MSIH class (Fig. 4B). Consistent with previously described results, generated edits toward MSIH showed a progressive emergence of features associated with MSI, including the disappearance of glandular architecture, reflected in a more solid growth pattern and increased intratumoral lymphocytes, whereas the microsatellite stable direction restored gland formation and reduced mucin content. These bidirectional transitions demonstrate that MoPaDi modifies tissue morphology in a class-specific and interpretable manner, confirming that the model has captured histologically relevant representations of MSI status.

### Style–morphology analysis disentangles staining from structural effects

**Figure 5.**
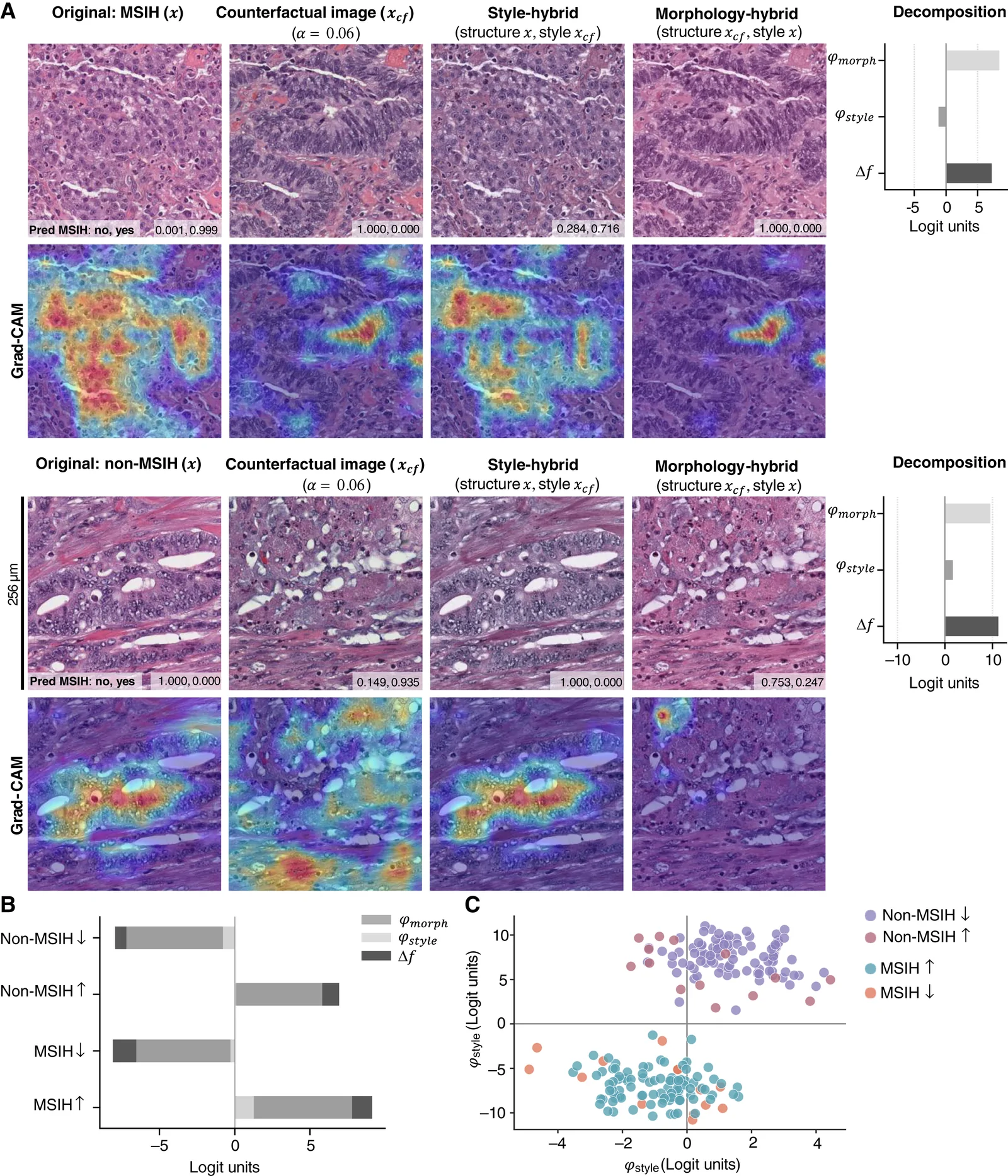
Style–morphology decomposition for disentangling structural and staining effects on MSI prediction changes. **A,** Representative examples of counterfactual manipulation between MSIH and non-MSIH classes. For each original image *x* and its counterfactual *x*_*cf*_, style-hybrid (*x*_*style*_) and morphology-hybrid (*x*_*morph*_) images were generated using Vahadane stain transfer. Each hybrid isolates the effect of either stain or morphology while controlling for the other. The right-hand bars show the Shapley-style decomposition of the logit change (Δ*f*) into stain (*φ*_*style*_) and morphology (*φ*_*morph*_) contributions, demonstrating that morphologic differences dominate the model’s predictions. Grad-CAM visualizations below provide region-level attribution under MIL. In contrast, MoPaDi produces class-directed “what-if” edits that offer a complementary view of candidate morphologic and style changes associated with prediction shifts. Scale bar applies to all images within the panel. **B,** Decomposition results across test-set patients, showing median contributions of *φ*_*style*_, *φ*_*morph*_, and total (Δ*f*) for manipulations toward (↑) and away from (↓) each class. **C,** Scatter plot of morphology versus style contributions per patient, illustrating consistent dominance of morphologic effects across both manipulation directions and classes.

To disentangle which visual aspects of the tissue drive the model’s predictions, we performed a style–morphology decomposition of real and counterfactual image pairs. This analysis aimed to separate the overall prediction change (Δ*f*) into contributions from staining style and tissue morphology, thereby quantifying their relative influence on model decision-making (Fig. 5).

We aggregated the decomposition results across all counterfactual tiles to quantify the overall contributions of style and morphology to the MSIH logit, as class transitions often include changes in staining. Across all colorectal cancer test-set patients (*N* = 108), manipulations toward the MSIH class yielded a median logit increase (Δ*f*) of +8.9 (IQR 7.2–10.1). The morphology-related component (*φ*_*morph*_) accounted for most of this shift (median +7.4; IQR 5.7–8.7), whereas the stain-related component (*φ*_*style*_) was comparatively small (+1.1; IQR 0.4–2.4). Morphologic features dominated in all patients, and the two components were generally reinforcing (conflict fraction close to 0). These findings indicate that model decisions for MSIH status are primarily driven by morphologic cues rather than staining variation.

We next analyzed counterfactual manipulations toward the non-MSIH class, aggregating decomposition results across all test-set patients. This analysis quantifies how the model’s MSIH evidence decreases when images are driven toward non-MSIH–like appearances. The manipulation resulted in a median logit change (Δ*f*) of −7.6 (IQR −9 to −6.3), indicating a consistent reduction of MSIH evidence. Again, the morphology-related component accounted for the majority of this decrease (median −6.6; IQR −8.2 to −5), whereas the stain-related component contributed only modestly (median −0.7; IQR −1.9 to −0.1). Morphologic effects dominated in all patients (morphology-dominant fraction = 1), with a small subset (conflict fraction of 0.2) showing opposing effects between style and morphology.

To further investigate whether decomposition patterns depended on ground-truth MSIH status, we stratified patients into MSIH and non-MSIH groups and repeated the aggregation separately. Across 109 colorectal cancer WSIs, bidirectional decomposition showed consistent morphologic dominance in both groups. Manipulations toward MSIH increased model confidence [Δ*f* = +9.1 (IQR 7.8–10.5)], primarily through morphology-related components [*φ*_morph_ = +7.8 (6.3–8.9)] with minor staining influence [*φ*_style_ = +1.3 (0.6–2.6)]. Conversely, transitions away from MSIH reduced confidence [Δ*f* = −8.1 (−10 to −6.3)], again morphology-driven [*φ*_morph_ = −6.5 (−9.1 to −5.1)]. For true non-MSIH samples, manipulations toward non-MSIH and away from non-MSIH showed symmetric logit shifts [+6.9 (5.4–9) and −7.9 (−9.2 to −6.7), respectively], both dominated by morphologic effects [*φ*_morph_ = 5.8 (4–8.3) and −7.2 (−8.3 to −6.1), respectively]. Morphology dominated in nearly all patients (fraction ≈1), and style–morphology conflicts were infrequent (0–0.3).

Together, these results show that morphologic structure is the primary driver of MSIH discrimination, with staining variations exerting a minor, sometimes opposing, influence.

### Evaluation across additional tumor types reveals characteristic morphologic changes

**Figure 6.**
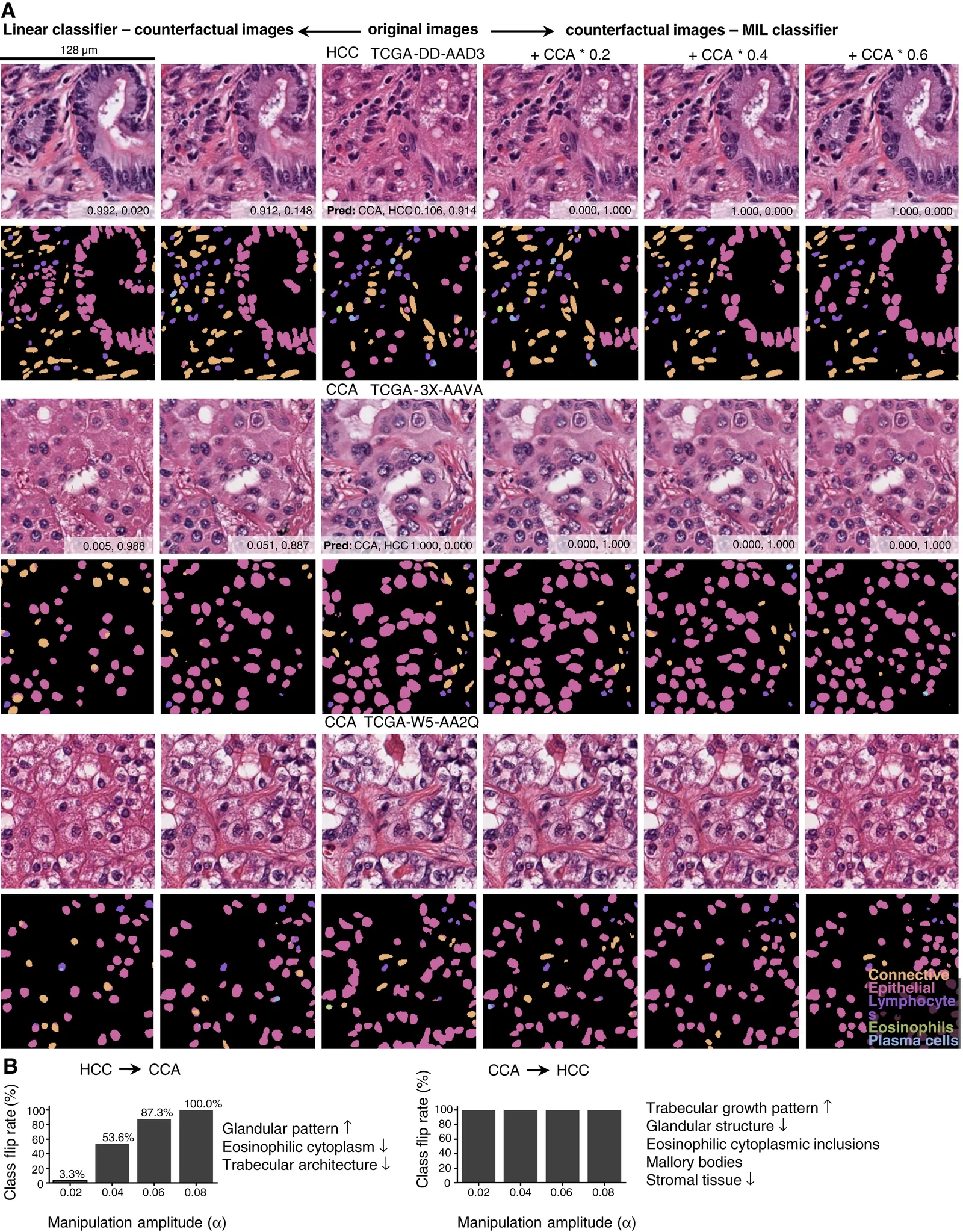
Counterfactual image examples generated for the liver cancer type classifier [hepatocellular carcinoma (HCC) vs. cholangiocarcinoma (CCA)]. **A,** Representative examples of counterfactual transitions generated with two approaches (linear and MIL) and a comparison of corresponding segmentation masks. **B,** Counterfactual image generation effectiveness, measured as the percentage of generated images predicted as the opposite class across varying manipulation amplitudes. HCC-to-CCA required higher amplitudes to achieve similar results, which likely reflects class imbalance in the dataset. Scale bar applies to all images within the panel.

We next evaluated MoPaDi across additional cancer types to assess whether the framework generalizes beyond colorectal cancer. First, a logistic regression classifier trained on MoPaDi features distinguished two hepatobiliary cancer types: hepatocellular carcinoma and cholangiocarcinoma, in the TCGA pan-cancer dataset with an AUC of 0.90 ± 0.01 (95% CI) and an AP of 0.57 ± 0.03 on the held-out test set. The lower AP is attributed to class imbalance in the dataset, with only 10% of patients diagnosed with cholangiocarcinoma (Supplementary Table S2). Patient-level averaging increased the AUC to 0.94 ± 0.10. A qualitative analysis of the generated counterfactual images revealed that the model captured representative morphologic features for both classes [Fig. 6A (left)]. Hepatocellular carcinoma tiles manipulated to resemble cholangiocarcinoma displayed a more glandular pattern, reduced eosinophilic cytoplasm, and less trabecular architecture. Meanwhile, cholangiocarcinoma tiles manipulated to resemble hepatocellular carcinoma displayed a more trabecular growth pattern with loss of glandular structure, eosinophilic cytoplasmic inclusions, possible Mallory bodies, and reduced stromal tissue. These counterfactual changes support that the model captures hepatobiliary cancer characteristics.

Given the complexity of weakly supervised WSI tasks, we assessed classification with attention-based MIL of different cancer types: lung cancer (lung adenocarcinoma vs. LUSC), breast cancer [invasive lobular carcinoma (ILC) vs. invasive ductal carcinoma (IDC)], and hepatocellular carcinoma versus cholangiocarcinoma. For the latter task, integrating MIL marginally improved predictive performance over the tile-based linear approach, achieving a mean AUC of 0.96 ± 0.01 and an AP of 0.82 ± 0.07 in the fivefold cross-validation. The MIL classifier was further evaluated on an independent hepatobiliary cohort from Charité (496 patients; cohort characteristics are provided in Supplementary Table S3), achieving an AUC of 0.85. Top tiles displayed distinctive features: original hepatocellular carcinoma tiles showed cells arranged in sheets or trabecular patterns with lipid droplets, lacking glandular structures and fibrous stroma. In contrast, original cholangiocarcinoma tiles exhibited glandular structures and more fibrous stroma (Supplementary Fig. S13A). Counterfactual images guided by the MIL classifier resembled those generated using the linear approach with tile-level logistic regression (Fig. 6A). Increasing manipulation amplitude (α) led to higher opposite-class predictions, with 100% of generated images being predicted as the opposite class at α = 0.08 (Fig. 6B). The cell type composition did not differ significantly between counterfactual and real images, indicating that counterfactual edits preserved underlying tissue composition (Supplementary Fig. S13A, statistical details in Supplementary Table S6).

**Figure 7.**
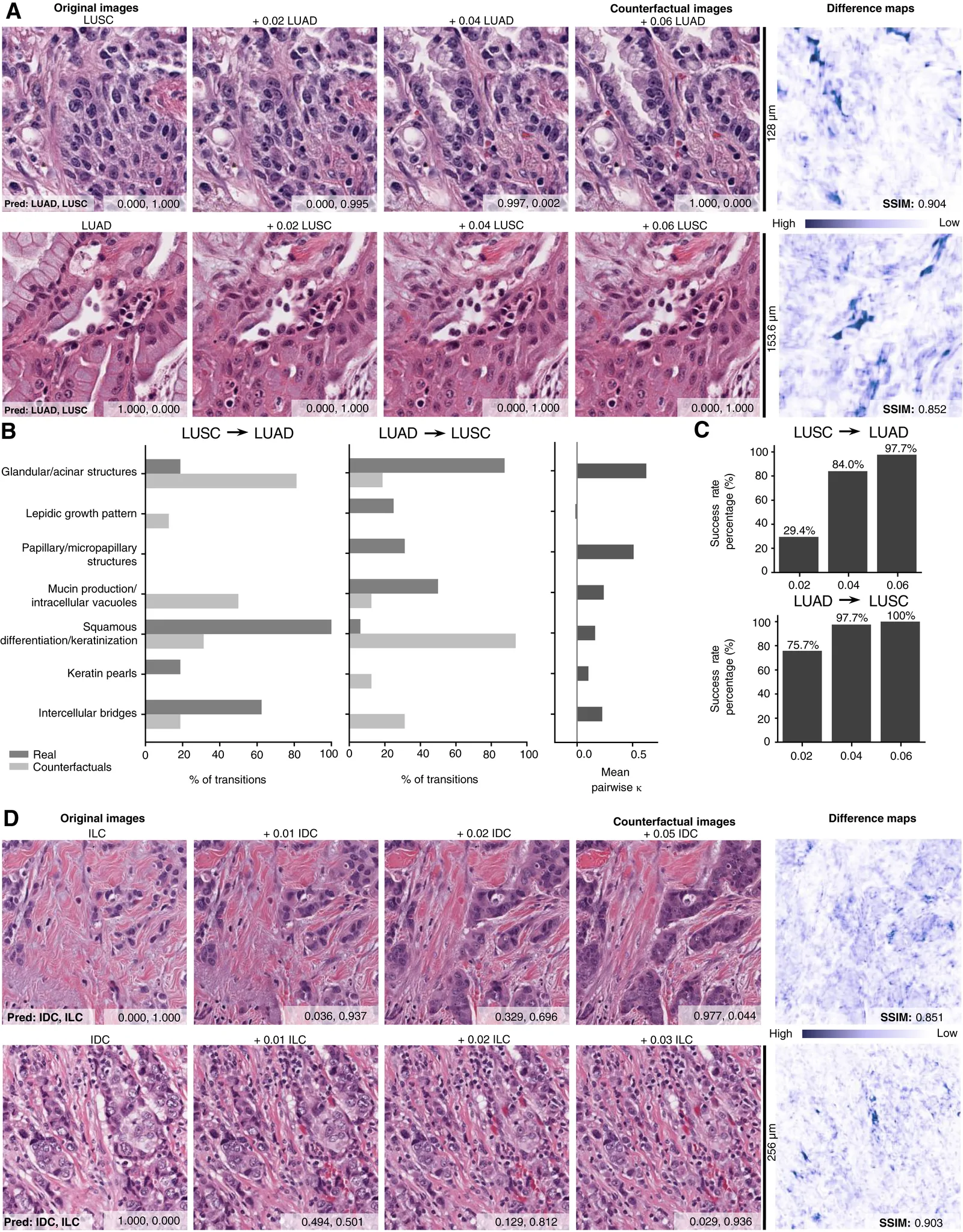
Counterfactual image examples generated for the lung and breast cancer–type classifiers. **A,** Representative examples of LUSC tile transitioning to its counterfactual lung adenocarcinoma (LUAD) image and *vice versa*. **B,** Morphologic feature prevalence in original and counterfactual image pairs (*N* = 32 transitions; 16 tiles for each class). Horizontal bars show the percentage of image pairs in which at least one of three raters (three board-certified pathologists) identified each morphologic feature as present in the original (dark gray) or counterfactual (light gray) image. Right, Mean pairwise inter-rater agreement (Cohen κ) per feature, computed across all pairs and both directions combined. **C,** Counterfactual image generation effectiveness, measured as the percentage of generated images predicted as the opposite class across varying manipulation amplitudes. **D,** Representative examples of ILC tile transitioning to its counterfactual IDC image and *vice versa*. Difference maps display pixelwise differences between the original and the synthetic tile. Scale bar applies to all images within the panel unless otherwise indicated.

For lung cancer typing, an MIL classifier distinguished lung adenocarcinoma from LUSC with a mean AUC of 0.91 ± 0.01 (an improvement of 0.10 over the linear method) and an AP of 0.89 ± 0.02. When evaluated on independent frozen-section lung cancer cohorts from CPTAC comprising 1,534 WSIs from 333 patients, it achieved an AUC of 0.77. Pathologist review showed that counterfactual transitions were accompanied by systematic shifts in expected histomorphologic features (Fig. 7A). In squamous cell carcinoma to adenocarcinoma transitions, raters identified the emergence of glandular or acinar architecture and mucin production or intracellular vacuoles, whereas squamous features such as squamous differentiation or keratinization, keratin pearls, and intercellular bridges were reduced (Fig. 7B). However, some counterfactual tiles retained features of LUSC atypical for lung adenocarcinoma, such as prominent dyskaryotic changes, nuclear molding, and residual squamous differentiation (Supplementary Fig. S14A), suggesting that the model may prioritize other features when making classification decisions. Conversely, transitions from adenocarcinoma to squamous cell carcinoma showed loss of glandular and papillary/acinar features, together with increased squamous differentiation/keratinization, with keratin pearls and intercellular bridges also appearing more frequently in the counterfactual images (Supplementary Fig. S14B). Counterfactual images maintained largely stable cell type compositions, with minor differences observed toward squamous cell carcinoma (Supplementary Fig. S14C; statistical details in Supplementary Table S7). Counterfactual image generation effectiveness remained high across both classes, with 98.8% of counterfactual images at amplitude α = 0.06 being predicted as the opposite class (Fig. 7C). Taken together, these results indicate that MIL classifier-guided counterfactual image generation produced edits that aligned with known morphologic differences between lung cancer types.

Finally, we investigated whether meaningful morphologic features could be identified in counterfactuals for the classification of IDC and ILC in the TCGA-BRCA cohort. The trained classifier reached a mean AUC of 0.86 ± 0.02 and an AP of 0.64 ± 0.04. To assess generalizability, we evaluated the classifier on the independent breast cancer cohort from CPTAC (frozen tissue; 323 WSIs from 100 patients), achieving an AUC of 0.70. In ILC-to-IDC counterfactual transitions [Fig. 7D (top); Supplementary Fig. S13B (top)], the most consistently observed changes were increased hyperchromasia, identified by both raters in 62.5% of examples, and atypia, identified by both raters in 50% of examples. Other notable features included a shift toward fewer single-cell arrangements without clear glandular pattern formation, noted in 25% to 37.5% of examples across raters, larger tumor cells in 37.5% of examples, and tumor cell arrangement in larger nests in 25% of examples. Some top tiles contained only muscle with single tumor cells, making feature assessment not feasible but potentially reflecting the diffusely infiltrative nature of these neoplasms. Meanwhile, IDC-to-ILC counterfactual transitions [Fig. 7D (bottom); Supplementary Fig. S13B (bottom)] lacked glandular architecture in 87.5% of examples, whereas less prominent nucleoli and single-cell arrangements of tumor cells were observed in all examples. Cell type quantification reflected an asymmetry between counterfactual directions: IDC-to-ILC counterfactuals partially approached the composition of real ILC, whereas ILC-to-IDC counterfactuals remained compositionally distinct from real IDC, with higher connective and lower epithelial content (Supplementary Fig. S13C; statistical details in Supplementary Table S8). Although the generated IDC-to-ILC images seemed to be plausible ILC representations (Supplementary Fig. S12) and approximately 97% of counterfactuals were predicted as the opposite class at the highest manipulation amplitude (Supplementary Fig. S13D), the top-contributing tiles for which counterfactuals were generated often lacked characteristic IDC features. Overall, despite the classifier’s moderate performance and inconsistent features in top tiles, we identified distinct morphologic characteristics of ILC and IDC, indicating that the classifier captured meaningful distinctions between these cancer types.

## Discussion

Throughout this study, we developed MoPaDi, a diffusion autoencoder-based framework for generating counterfactual images, and extended it with MIL to handle WSIs. MoPaDi demonstrates how diffusion models can extend computational pathology from passive description toward model-based virtual perturbation analyses. Our framework enables pathologists to explore model-linked “what if” questions through counterfactual analysis. Although these edits do not imply biological causality, they provide model-internal simulations of morphologic change and enable auditing of trained classifiers.

The effectiveness of our approach was evaluated across multiple experimental scenarios spanning several tumor types, providing quantitative and qualitative evidence for the plausibility of the generated images using several orthogonal measures. Diffusion autoencoders achieved excellent image reconstruction quality, as reflected by MS-SSIM and MSE. However, such pixel-level metrics provide only partial information about reconstruction fidelity and do not fully capture clinically relevant subcellular or histomorphologic details. We therefore complemented them with blinded expert evaluation, which showed that MoPaDi-generated counterfactual images were often difficult for pathologists to distinguish from real histologic samples. We further found that MoPaDi captures biologically plausible patterns. For example, overall cell type trends in generated colorectal cancer images aligned with established tumor and adjacent normal tissue microenvironment characteristics (46). Tumor tiles showed high epithelial cell abundance, particularly in normal-to-tumor transitions, suggesting that the model captured epithelial abundance as a feature associated with tumor predictions, consistent with tumor biology (47). Similarly, for liver and lung tumor typing, the model generated known features, including trabecular-to-glandular transitions and differences in fibrous stroma in liver samples (48–50).

We also extended our analysis to molecular biomarkers by investigating MSI status in colorectal cancer. Because the MSI classifier achieved only moderate performance compared with foundation model–based approaches (3), we interpret the MSI counterfactuals as model-derived hypotheses and as an audit of learned features rather than direct biological readouts. Nevertheless, MoPaDi revealed classifier-associated patterns overlapping with reported MSI morphology. Even with a limited set of counterfactual transitions from top-contributing tiles, pathologists identified characteristic features, such as signet-ring cells, mucinous and medullary patterns, inflammatory infiltration, and poor differentiation, consistent with prior literature (51–55). Previous GAN-based work has also explored MSI-associated morphology using latent manipulation and class activation mapping–guided image transformations (16), whereas MoPaDi uses diffusion autoencoder–based counterfactual editing to generate progressive classifier-guided transitions while preserving much of the original tissue context. This design enables inspection of feature changes along a continuous counterfactual trajectory within the same tissue region, facilitating the attribution of classifier-associated morphology.

Because staining variation can bias digital pathology models (56–58), we used MoPaDi for an approximate style–morphology decomposition. In the MSI classifier setting, the analysis suggested that model decisions were predominantly morphology-driven, whereas stain effects were smaller or sometimes opposing. Because Vahadane-based stain transfer may not perfectly separate stain from tissue structure, we interpret this decomposition as supporting evidence rather than definitive proof of complete disentanglement. Nevertheless, the framework may help identify potential staining biases at the per-image level and provide a useful assessment of style-related confounders in digital pathology models. More broadly, MoPaDi visualizes model-internalized signals, and its interpretation therefore remains dependent on classifier quality and robustness. Consequently, when applied to a strong classifier, counterfactuals may support biologically meaningful hypothesis generation. In contrast, for lower-performing or partially confounded classifiers, counterfactuals may primarily serve to expose shortcut learning, site-specific bias, staining dependence, or incompletely learned morphology.

Finally, to the best of our knowledge, MoPaDi is the first generative model to operate within an MIL framework to generate counterfactual explanations for biomarkers that are defined only at the level of slide annotations. This capability is important because many molecular and clinical features in computational pathology are available only at the whole-slide level. By combining diffusion models with MIL, we enable the interpretation of weakly supervised learning problems common in clinical practice.

Our approach has several important limitations. Results are currently limited to tile-level analysis and require careful validation by domain experts. Although MoPaDi was evaluated across several tumor types, the present study does not establish broader generalizability across cancer histologies. In addition, although the framework is not inherently restricted to a specific magnification, we did not systematically evaluate scale effects. From a practical perspective, each dataset currently requires training a custom autoencoder model, which is computationally intensive and time-consuming. Although the model typically generates realistic images, rare counterfactuals showed limitations in subtle histomorphologic details, including occasional examples in the colorectal cancer normal mucosa versus tumor epithelium task, with incomplete basement membrane delineation in tumor-to-normal mucosa transitions or limited nuclear hyperchromasia in normal-to-tumor transitions. Such examples may also suggest that the classifier relied more strongly on broader architectural cues than on fine-grained structural features or cytologic detail. Occasional imperfections were more apparent in tiles with sparse tissue or substantial background, in which limited morphologically informative content may constrain faithful counterfactual editing. Additionally, although the overall cell type distributions broadly aligned with known tumor biology, the model showed potential biases in generating certain cell types over others, highlighting the importance of expert validation when interpreting generated transitions. We also did not systematically assess MoPaDi’s behavior in the presence of slide artifacts such as folds, air bubbles, or scanner-related noise. Because the framework is model-faithful, artifacts irrelevant to the classifier may simply be retained as incidental image content, whereas artifact-related cues learned by the classifier could be preserved or reinforced during counterfactual generation, thereby representing a potential source of bias. Future work could address these limitations by extending validation to additional tumor entities, evaluating multiscale workflows, and integrating pathology foundation models with diffusion-based counterfactual generation to improve both predictive performance and biological accuracy while maintaining the advantages of high-quality image synthesis and selective feature manipulation.

Looking forward, this work opens new possibilities for computational pathology. By enabling virtual experimentation, tools like MoPaDi could help researchers prescreen hypotheses before wet-lab experimentation. Additionally, these methods could provide new insights into morphologic features that correlate with outcomes, potentially revealing previously unknown biological mechanisms. An important next step will be to evaluate MoPaDi in formal user studies against established explainability methods, such as attention heatmaps or Grad-CAM, to assess whether counterfactuals improve the efficiency of expert review. As these methods continue to evolve, they may become useful tools in cancer research, complementing traditional experimental approaches and aiding hypothesis generation in cancer biology.

## Supplementary Material

Figure S1 Colorectal cancer tissue classes and classifier performance

Figure S2 MIL transformer architecture for patient-level classification

Figure S3 Reconstruction quality across datasets

Figure S4 Synthetic image samples accross models

Figure S5 Tissue-type counterfactual examples for additional classes

Figure S6 Additional tumor to normal colon mucosa counterfactual transitions

Figure S7 MSIH to nonMSIH counterfactuals with cell segmentation

Figure S8 nonMSIH to MSIH counterfactuals with cell segmentation

Figure S9 Counterfactuals for borderline vs. top-contributing MSI tiles

Figure S10 Cellular/morphological changes in MSIH counterfactual transitions

Figure S11 External foundation-model validation of MSI counterfactuals

Figure S12 Perceptual realism expert study

Figure S13 Counterfactual results for liver and breast cancer types

Figure S14 Counterfactual results for the lung cancer type classifier

Supplementary Tables Supplementary Tables S1-8

## Data Availability

Digital histopathology WSIs from TCGA, together with the clinical data used in this study, are available at https://portal.gdc.cancer.gov and https://cbioportal.org. NCT-CRC-HE-100K and CRC-VAL-HE-7K (1) are publicly available at https://zenodo.org/records/1214456. Curated histology image patches from uniform tumor regions of TCGA WSIs are available at https://zenodo.org/records/5889558. CPTAC WSIs are available through The Cancer Imaging Archive (https://www.cancerimagingarchive.net); associated clinical and molecular data are available through the NCI Genomic Data Commons Portal. The external validation cohort from Charité – Universitätsmedizin Berlin is not publicly available because it contains patient data subject to institutional and ethical restrictions.

## Code Availability

MoPaDi code and instructions for downloading trained models are publicly available on GitHub and Hugging Face at https://github.com/KatherLab/mopadi and https://huggingface.co/KatherLab/MoPaDi.
