# Supplementary material for: Counterfactual Diffusion Models Provide Interpretable Explanations of Artificial Intelligence Models in Pathology
Source: Cancer Res. 2026 Jun 17;86(17):4414–33. doi: 10.1158/0008-5472.CAN-25-5645 (PMC13535314; doi:10.1158/0008-5472.CAN-25-5645)
Supplement: Supplementary file 1 [file can-25-5645_figure_s10_suppsf10.pdf]

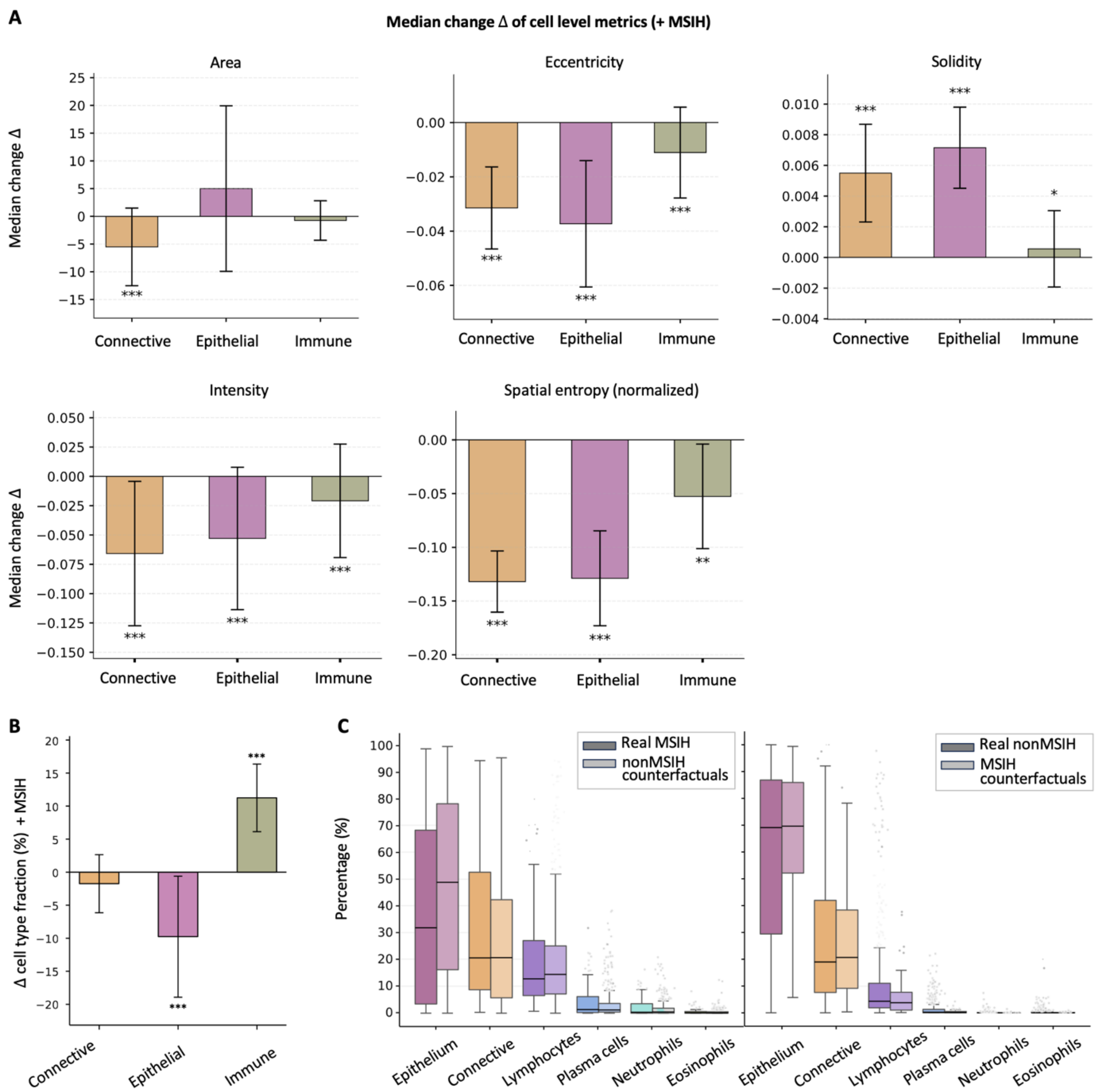

Figure S10. Cellular composition and morphological alterations associated with MSIH counterfactual transitions in TCGA CRC. (**A**) Median changes (Δ) in cell-level morphological metrics for counterfactual manipulations toward MSIH. **(B**) Change in cell-type fractions across connective, epithelial, and immune cell types when manipulating toward MSIH. MSIH counterfactuals show a significant increase in immune fraction and a reduction in epithelial fraction ($p < 0.001$, Wilcoxon paired test). (**C**) The distribution of cell-type percentages in: (**left**) real MSIH ($N = 65$) and nonMSIH counterfactual tiles ($N = 475$), (**right**): real nonMSIH tiles ($N = 475$) and counterfactual tiles ($N = 65$). Boxes/whiskers summarize tile-level values; gray dots are individual outlier tiles. Statistical inference was performed at the patient level (per-patient mean percentage). No cell type showed a statistically significant difference after multiple-testing correction across six tests; full statistics (p, adjusted p, Cliff's δ with 95% CI) are reported in Supplementary **Table S5.**
