# Supplementary material for: Counterfactual Diffusion Models Provide Interpretable Explanations of Artificial Intelligence Models in Pathology
Source: Cancer Res. 2026 Jun 17;86(17):4414–33. doi: 10.1158/0008-5472.CAN-25-5645 (PMC13535314; doi:10.1158/0008-5472.CAN-25-5645)
Supplement: Supplementary file 2 [file can-25-5645_figure_s11_suppsf11.pdf]

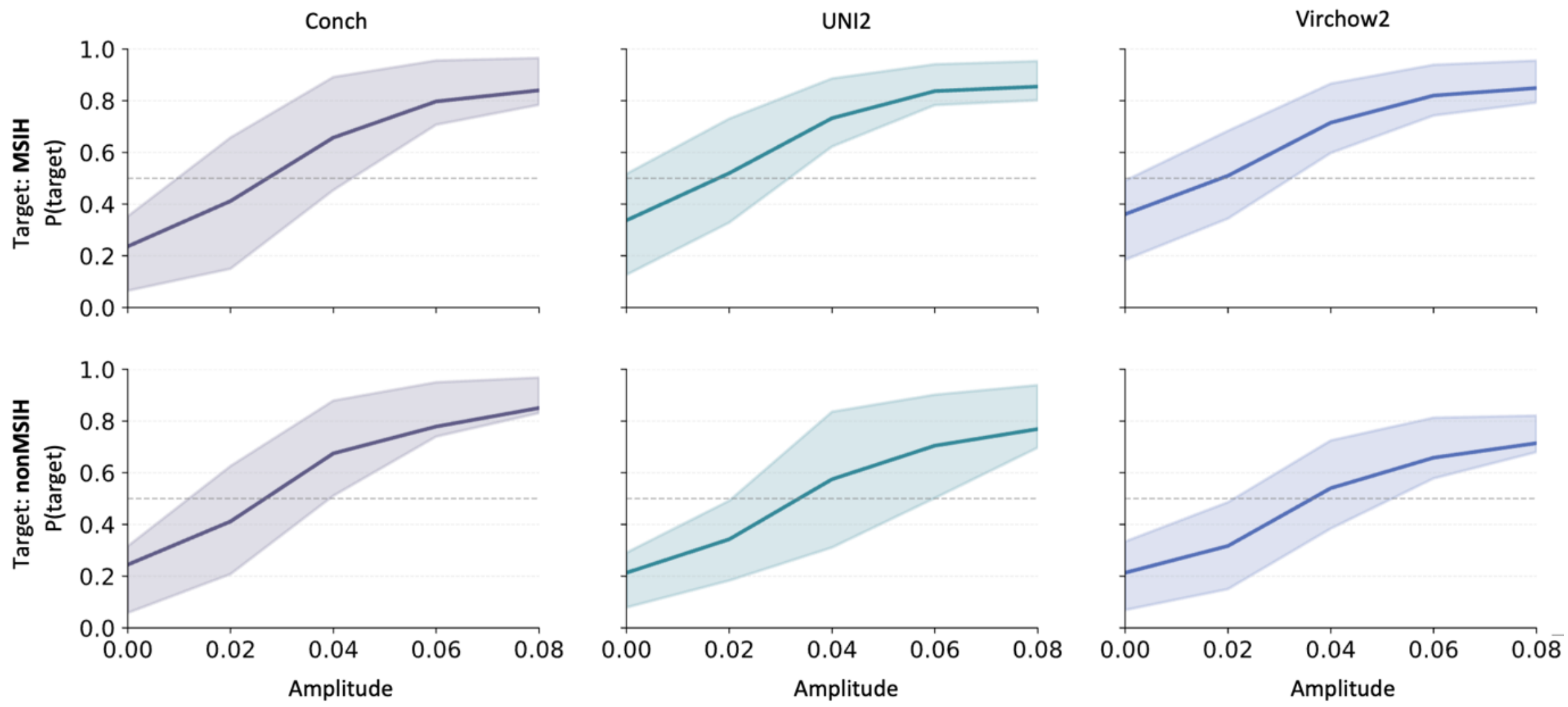

Figure S11. Validation of counterfactual transitions using independent histopathology classifiers. Prediction probabilities across counterfactual manipulation amplitudes (α) for three independent foundation model-based MSI classifiers (CONCH, UNI2, and Virchow2). Lines represent the mean predicted probability for the target class, and shaded regions denote the interquartile range across tiles. Across all models and directions, confidence shifts monotonically toward the intended class, demonstrating that MoPaDi counterfactual trajectories are supported by external classifiers and reflect biologically meaningful decision directions rather than model-specific artifacts.
