# Supplementary material for: Counterfactual Diffusion Models Provide Interpretable Explanations of Artificial Intelligence Models in Pathology
Source: Cancer Res. 2026 Jun 17;86(17):4414–33. doi: 10.1158/0008-5472.CAN-25-5645 (PMC13535314; doi:10.1158/0008-5472.CAN-25-5645)
Supplement: Supplementary file 3 [file can-25-5645_figure_s12_suppsf12.pdf]

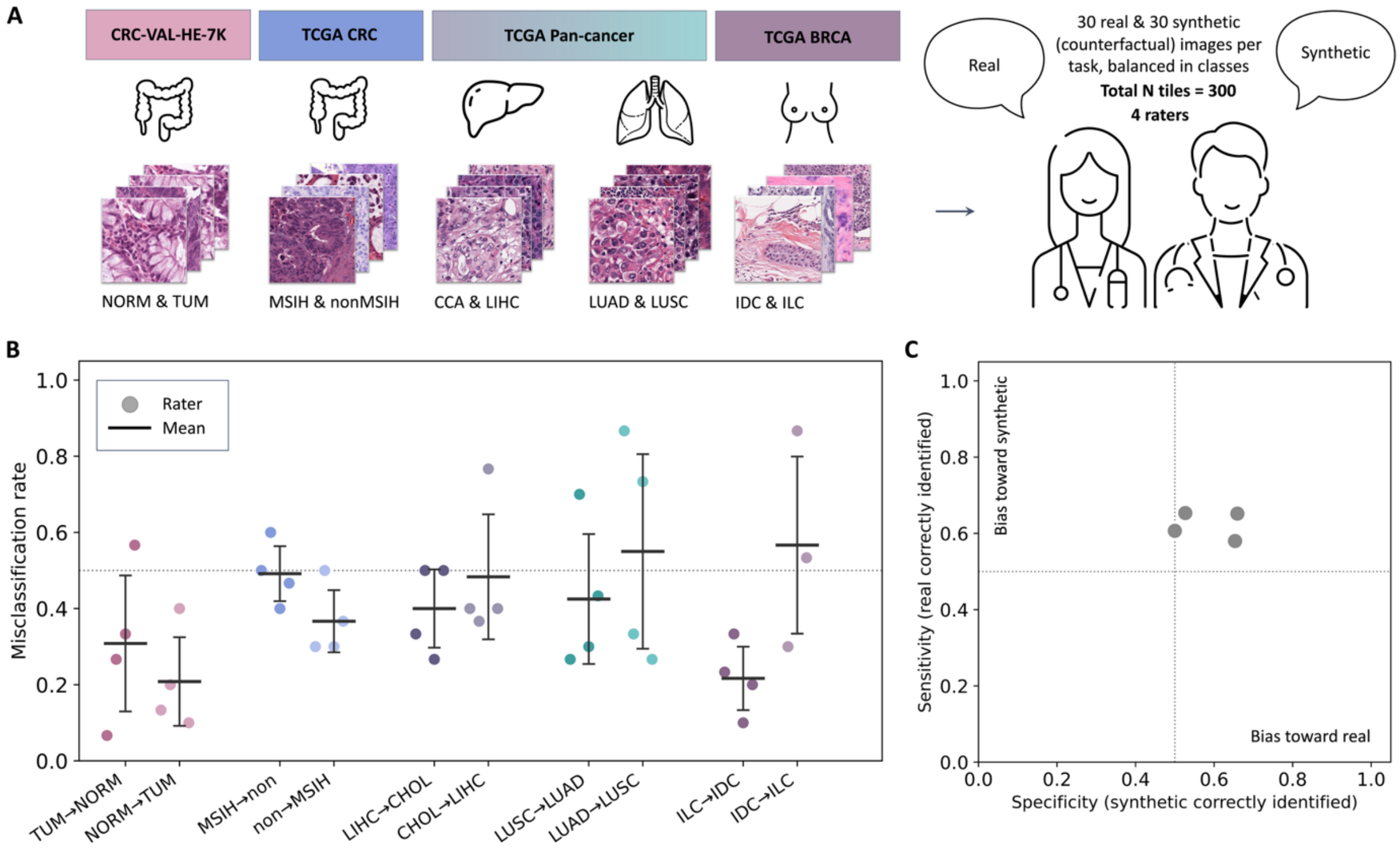

Figure S12. Overview and results of a tile-level user study evaluating real versus synthetic histopathology images to assess the perceptual realism of counterfactual images. (A) For each of the five evaluation tasks (one each for CRC-VAL-HE-7K, TCGA-BRCA, TCGA-CRC, and two for the TCGA Pan-cancer dataset), 30 real tiles (15 per class) and 30 corresponding synthetic (counterfactual) tiles were randomly selected (total N = 300). Four raters (three board-certified pathologists and one pathology trainee) independently reviewed the tiles and classified each one as either real or synthetic. (**B**) Misclassification rate for four raters across ten study sections, grouped by cancer type and counterfactual direction. Error bars show the standard deviation across experts. **(C)** Sensitivity and specificity of the raters for distinguishing real from synthetic histopathology tiles. Each dot represents one rater. Sensitivity reflects the proportion of real tiles correctly identified as real; specificity reflects the proportion of synthetic tiles correctly identified as synthetic. Experts tended to show higher sensitivity than specificity, consistent with a tendency to rate ambiguous tiles as real rather than synthetic. NORM, healthy colon mucosa; TUM, colorectal adenocarcinoma epithelium; IDC, invasive ductal carcinoma; ILC, invasive lobular carcinoma; CCA, cholangiocarcinoma; MSIH, microsatellite instability-high; LIHC, liver hepatocellular carcinoma; LUSC, lung squamous cell carcinoma; LUAD, lung adenocarcinoma.
