# Supplementary material for: Counterfactual Diffusion Models Provide Interpretable Explanations of Artificial Intelligence Models in Pathology
Source: Cancer Res. 2026 Jun 17;86(17):4414–33. doi: 10.1158/0008-5472.CAN-25-5645 (PMC13535314; doi:10.1158/0008-5472.CAN-25-5645)
Supplement: Supplementary file 4 [file can-25-5645_figure_s13_suppsf13.pdf]

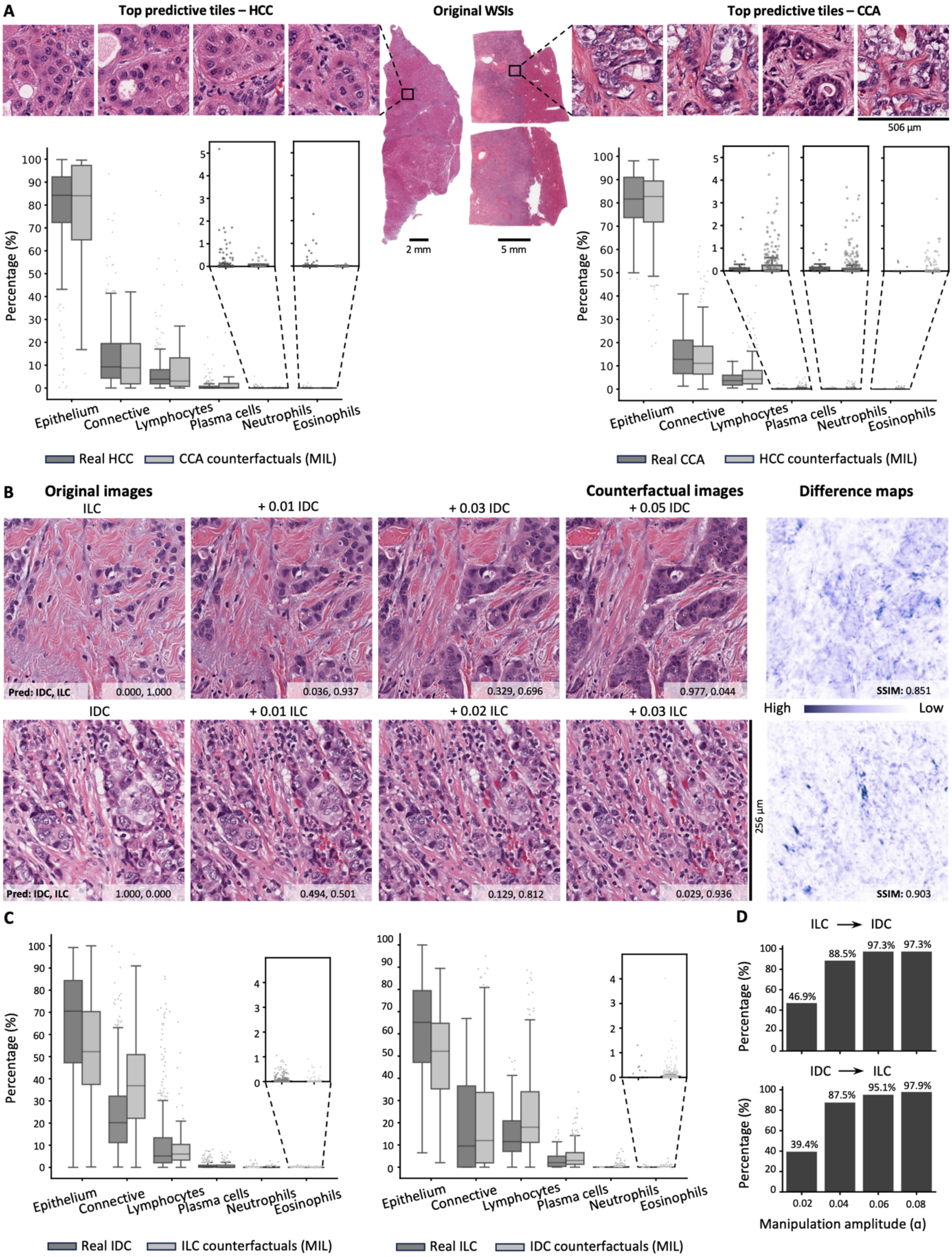

Figure S13. Supplementary results for the evaluation of counterfactuals for the classification of liver and breast cancer types. (**A**) Representative top-predictive tiles for hepatocellular carcinoma (HCC) and cholangiocarcinoma (CCA), and the distribution of different cell types in real and synthetic tiles. (**Left**) CCA tiles (31 tiles from 6 patients) and synthetic (counterfactual images of HCC tiles, i.e., the most manipu-

lated tiles, 276 tiles from 54 patients). No significant differences in cell-type percentages between the groups were observed after applying the Bonferroni correction for multiple comparisons, based on a two-sided Mann-Whitney U test (adjusted $p > 0.05$). (**Right**) Real HCC tiles (276 tiles from 54 patients) versus synthetic (counterfactual images of CCA tiles, i.e., the most manipulated tiles, 31 tiles from 6 patients). No significant differences in cell-type percentages between groups were observed. A full description of the statistical parameters, including p-values, is provided in Supplementary **Table S6**. (**B**) Representative example of invasive lobular carcinoma (ILC) tile transitioning to its counterfactual invasive ductal carcinoma (IDC) image, and vice versa. Difference maps display pixel-wise differences between the original and the synthetic tile. **(C)** The distribution of different cell types in real and synthetic images. (**Left**) IDC tiles ($N = 472$) versus synthetic (counterfactual images, i.e., tiles manipulated at $\alpha = 0.06$; $N = 113$). Statistically ($p < 0.001$) and practically (Cliff's delta > 0.147) significant differences in cell-type percentages were observed for epithelial and connective cells. (**Right**) Real ILC ($N = 115$) and synthetic ($N = 475$) tiles. Statistically significant differences in cell-type percentages were observed for all cell types, except epithelial and plasma cells. A full description of the statistical parameters, including *p*-values, is provided in Supplementary **Table S8.** (**D**) Quantitative evaluation of counterfactual image generation effectiveness. Bars show the percentage of successful manipulations (i.e., predicted class equals target class) across different manipulation amplitudes. Scale bar applies to all images within the panel unless otherwise indicated.
