# Supplementary material for: Counterfactual Diffusion Models Provide Interpretable Explanations of Artificial Intelligence Models in Pathology
Source: Cancer Res. 2026 Jun 17;86(17):4414–33. doi: 10.1158/0008-5472.CAN-25-5645 (PMC13535314; doi:10.1158/0008-5472.CAN-25-5645)
Supplement: Supplementary file 5 [file can-25-5645_figure_s14_suppsf14.pdf]

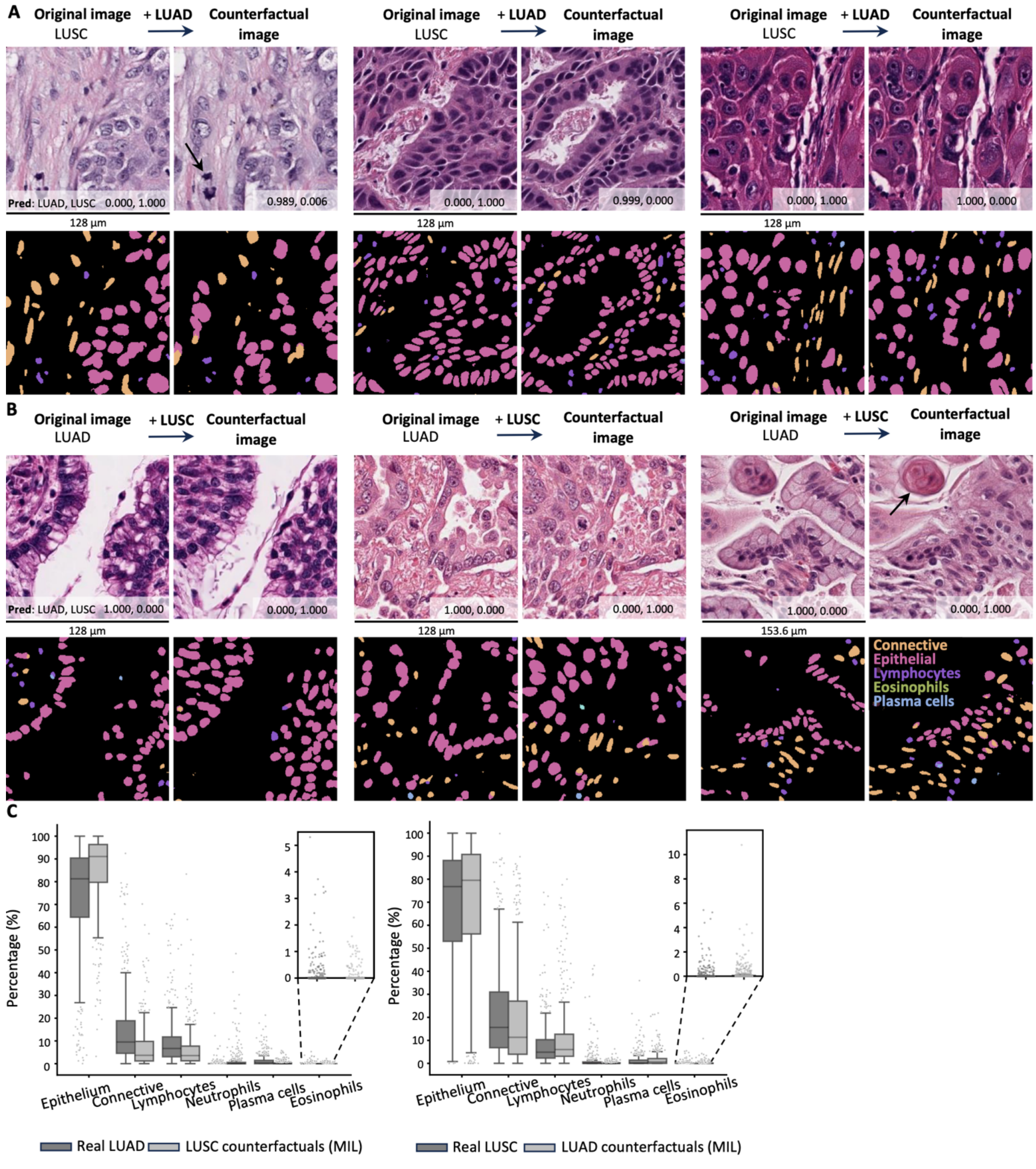

Figure S14. Results of counterfactual image generation for MIL-based lung cancer type classifier. (**A**) Representative examples of lung squamous cell carcinoma (LUSC) to lung adenocarcinoma (LUAD) transitions to counterfactual images. (**Left**) black arrow – remaining mitotic figure, which is more typical of LUSC than LUAD; (**middle**) glands with nuclear molding, which was a prominent feature in the original image and the counterfactual image as well; (**right**) remaining visible squamous differentiation in the counterfactual image with the solid morphology. (**B**) Representative examples of LUAD to LUSC transitions to counterfactual images. (**Left)** Remaining

glandular architecture with more cell layers in the counterfactual image; (**middle**) potentially visible intercellular bridges in the counterfactual image and a higher degree of nuclear atypia; (**right**) black arrow – potentially visible squamous pearl. (**C**) The distribution of different cell types in real and synthetic tiles. (**Left**) Real LUAD tiles (*N* = 470) and synthetic (counterfactual images of LUSC, i.e., the most manipulated tiles [$\alpha = 0.06$], where 98.8% of counterfactual images were predicted as the target class, *N* = 470). Statistically ($p < 0.001$) and practically (Cliff's delta > 0.147) significant differences in cell-type percentages were observed for all groups, except eosinophils. (**Right**) Real LUSC tiles (*N* = 470) versus LUAD counterfactual images (*N* = 470). Statistically significant differences in cell-type percentages were observed for neutrophils and plasma cells. However, these differences were practically significant only for plasma cells. A full description of the statistical parameters, including *p*-values, is provided in Supplementary **Table S7. (D)** Quantitative evaluation of counterfactual image generation effectiveness. Bars show the percentage of successful manipulations (i.e., predicted class equals target class) across different manipulation amplitudes.
