# Supplementary material for: Counterfactual Diffusion Models Provide Interpretable Explanations of Artificial Intelligence Models in Pathology
Source: Cancer Res. 2026 Jun 17;86(17):4414–33. doi: 10.1158/0008-5472.CAN-25-5645 (PMC13535314; doi:10.1158/0008-5472.CAN-25-5645)
Supplement: Supplementary file 6 [file can-25-5645_figure_s1_suppsf1.pdf]

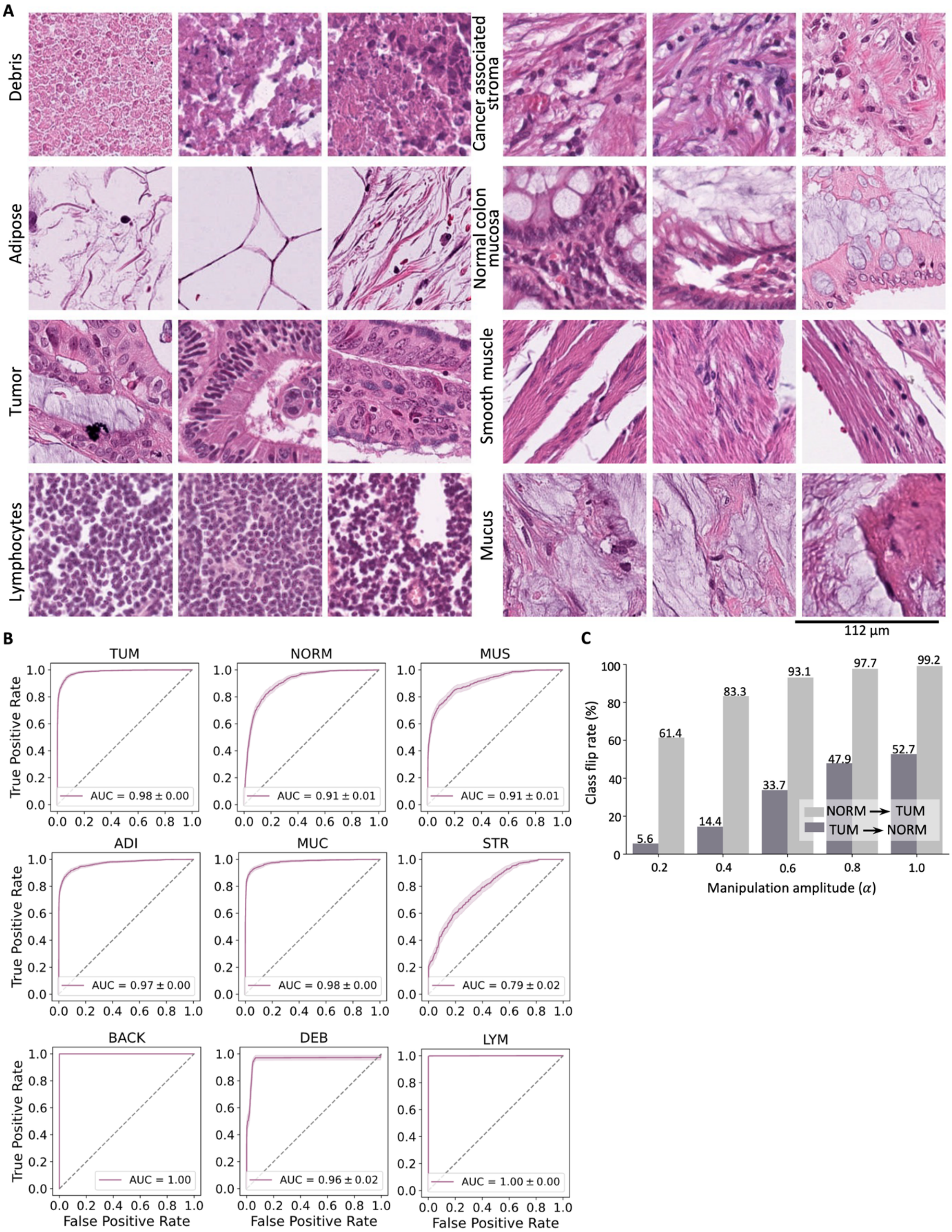

Figure S1. Representative examples of classes in the NCT-CRC-HE-100K dataset and receiver operating characteristic curves (ROC) evaluating the trained classifier's performance on the CRC-VAL-HE-7K dataset (*N* = 7,180 tiles). (A) NCT-CRC-HE-100K dataset contains nine classes: debris (DEB), adipose (ADI), tumor (i.e., dysplastic epithelium; TUM), lymphocytes (LYM), cancer-associated stroma (STR),

normal colon mucosa (NORM), smooth muscle (MUS), mucus (MUC), and background (not displayed; BACK). **(B)** Evaluation of the trained classifier's performance on the CRC-VAL-HE-7K shows good performance, with an area under the ROCs ranging from 0.79 ± 0.02 for the STR class to 1.00 ± 0.00 for the BACK and LYM classes. **(C)** Percentage of manipulated tiles predicted to be the target class as manipulation amplitude (α) increases. NORM to TUM manipulations require lower amplitudes to flip predictions compared to TUM to NORM, which is consistent with the classifier's performance, as the classification performance is lower for NORM.
