# Supplementary material for: Counterfactual Diffusion Models Provide Interpretable Explanations of Artificial Intelligence Models in Pathology
Source: Cancer Res. 2026 Jun 17;86(17):4414–33. doi: 10.1158/0008-5472.CAN-25-5645 (PMC13535314; doi:10.1158/0008-5472.CAN-25-5645)
Supplement: Supplementary file 7 [file can-25-5645_figure_s2_suppsf2.pdf]

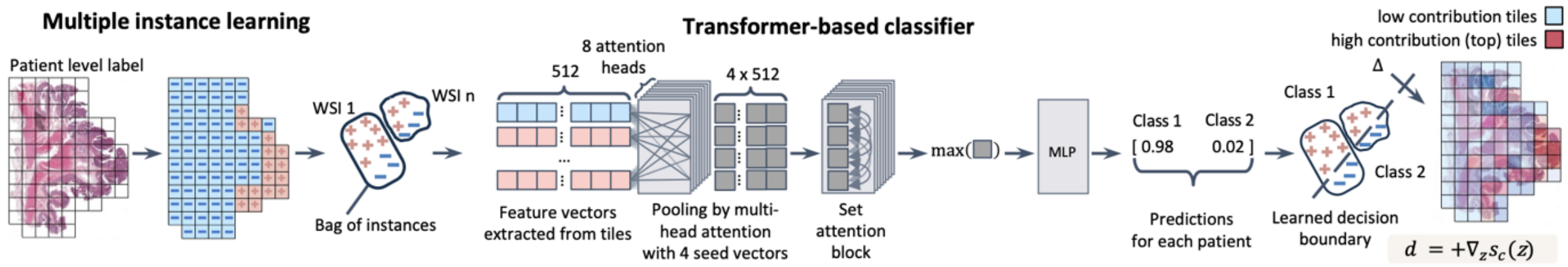

Figure S2. End-to-end multiple-instance learning (MIL) with a transformer for patient-level histopathology classification and tile-level interpretability. A whole-slide image (WSI) is partitioned into fixed-size tiles (e.g., 512 x 512 px at 0.5 micrometers per pixel for TCGA-CRC). Tiles from one or more WSIs of the same patient are grouped into a bag of instances and inherit the patient-level label (weak supervision; no per-tile labels). Each tile is encoded into a 512-dimensional feature vector using the pretrained MoPaDi encoder. Features are processed by the transformer: pooled multi-head attention (PMA) with 4 seed vectors and 8 heads aggregates the unordered set into a small set of pooled descriptors; a set attention block refines interactions among pooled descriptors. The architecture is permutation-invariant by design. A layer-norm precedes pooling. The pooled descriptors are collapsed to a single patient embedding via element-wise max over seeds. The patient embedding passes through a two-layer multilayer perceptron (MLP) to produce logits and class probabilities. The learned decision boundary separates patient bags in embedding space; the predicted class and confidence are shown. To identify tiles most influential for the model's decision, each tile feature vector is independently evaluated as a single-instance “bag”, yielding a per-tile class probability. Tiles are ranked by their individual softmax probability for the predicted class, and the highest-scoring (“top”) tiles are highlighted as high-contribution regions, while lower-scoring tiles are shown as low-contribution regions. This produces a tile-level importance map that reflects the tiles whose isolated features most strongly support the patient-level prediction, complementing the weakly supervised MIL framework. Top-contributing tiles are subsequently used to generate counterfactual images.
