# Supplementary material for: Counterfactual Diffusion Models Provide Interpretable Explanations of Artificial Intelligence Models in Pathology
Source: Cancer Res. 2026 Jun 17;86(17):4414–33. doi: 10.1158/0008-5472.CAN-25-5645 (PMC13535314; doi:10.1158/0008-5472.CAN-25-5645)
Supplement: Supplementary file 8 [file can-25-5645_figure_s3_suppsf3.pdf]

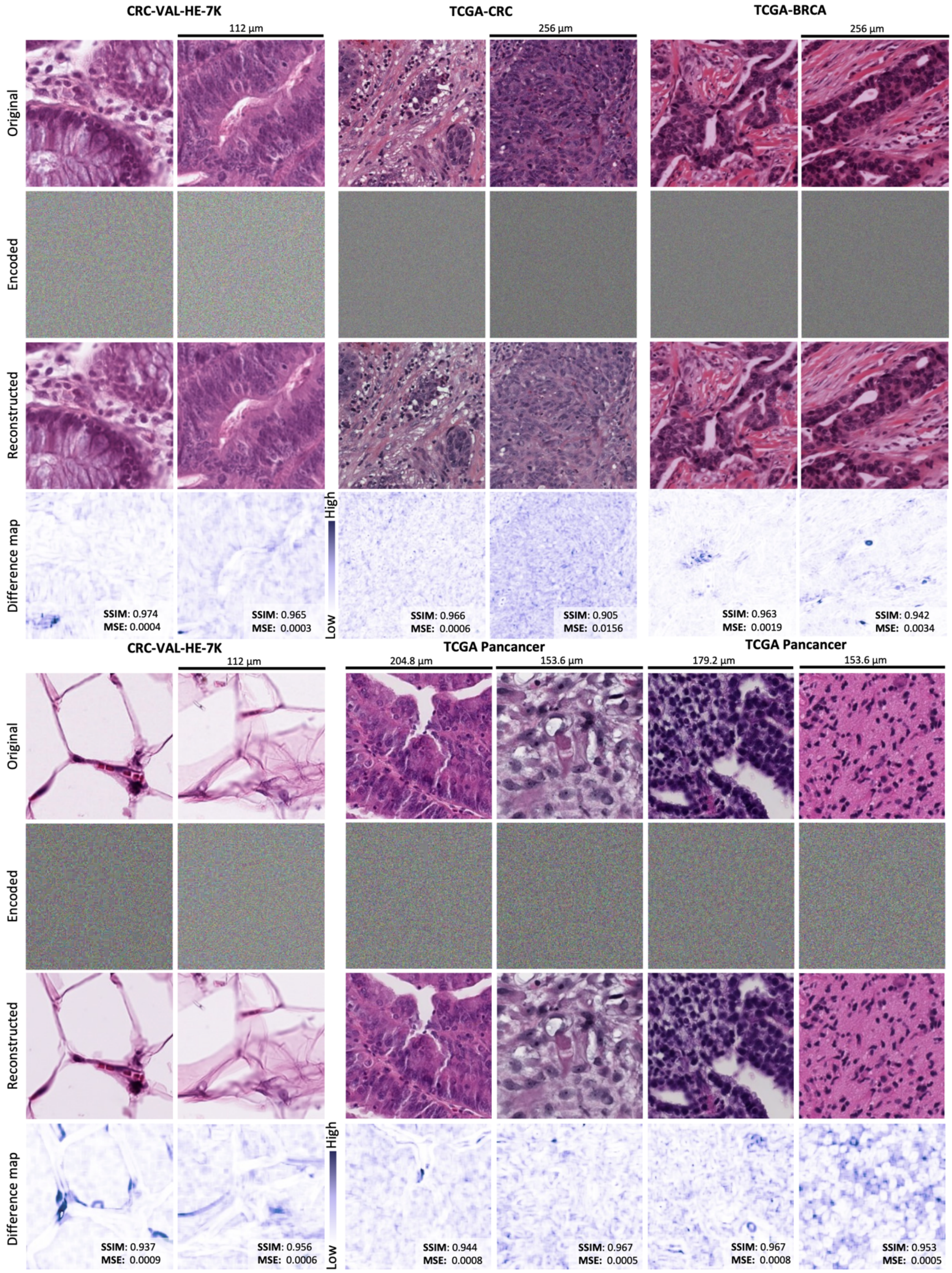

Figure S3. Representative examples from all datasets showing original images, their latent noise representations, and corresponding reconstructions. Random representative tiles from each dataset alongside their latent noise representations produced by the custom-trained diffusion autoencoder, followed by the corresponding reconstructed images. The model sometimes struggles with structure boundaries and white spaces. Difference images visually represent dissimilarity between the original

and reconstructed images (white regions indicate perfect similarity, while darker regions indicate dissimilarity) and are displayed alongside quantitative metrics of reconstruction quality.
