# Supplementary material for: Counterfactual Diffusion Models Provide Interpretable Explanations of Artificial Intelligence Models in Pathology
Source: Cancer Res. 2026 Jun 17;86(17):4414–33. doi: 10.1158/0008-5472.CAN-25-5645 (PMC13535314; doi:10.1158/0008-5472.CAN-25-5645)
Supplement: Supplementary file 9 [file can-25-5645_figure_s4_suppsf4.pdf]

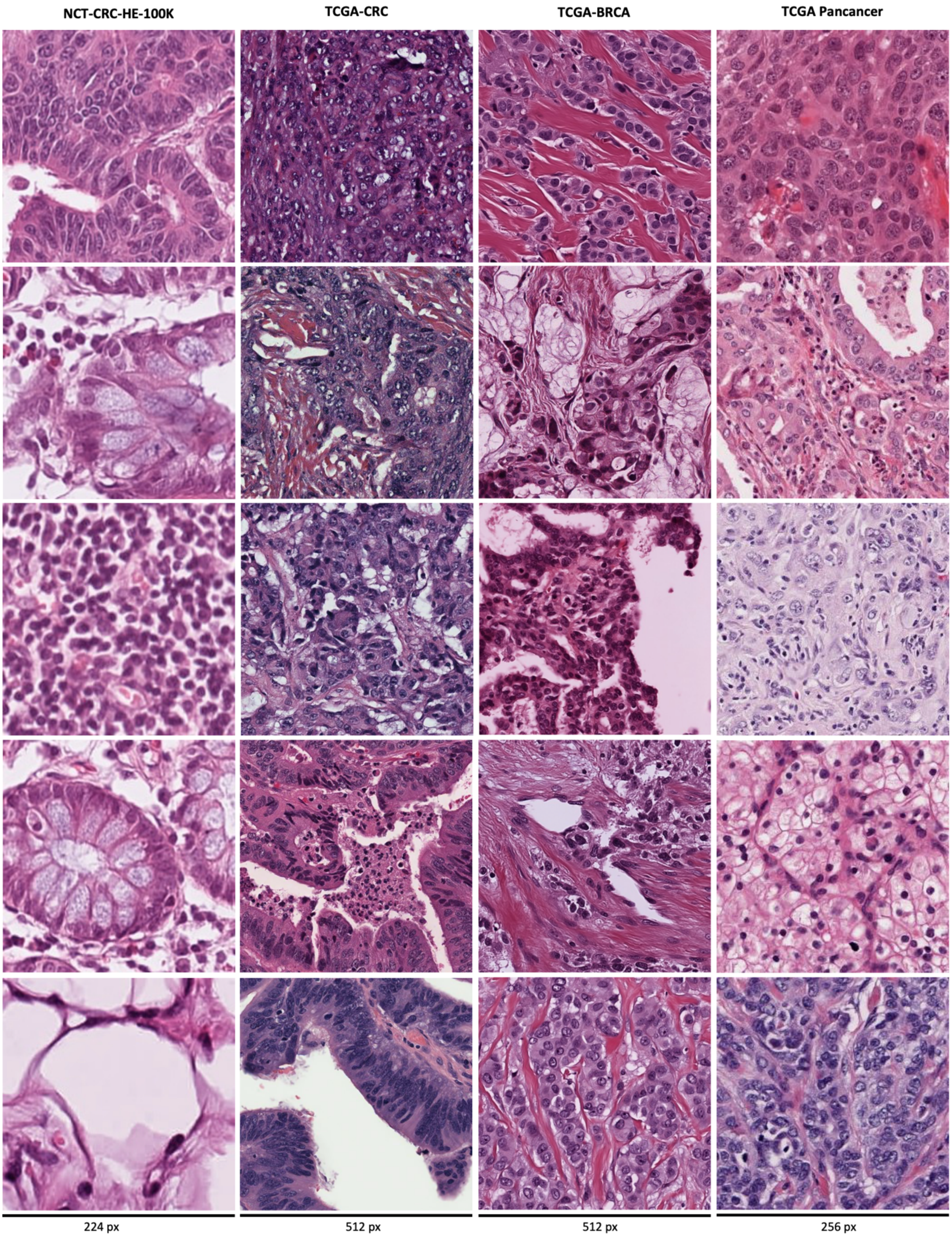

Figure S4. Randomly sampled representative examples of generated synthetic images with four trained models trained on different datasets: NCT-CRC-HE-100K, TCGA-CRC, TCGA-BRCA, and TCGA Pan-cancer. The scale bar corresponds to the image size used to train the generative model.
