# Supplementary material for: Counterfactual Diffusion Models Provide Interpretable Explanations of Artificial Intelligence Models in Pathology
Source: Cancer Res. 2026 Jun 17;86(17):4414–33. doi: 10.1158/0008-5472.CAN-25-5645 (PMC13535314; doi:10.1158/0008-5472.CAN-25-5645)
Supplement: Supplementary file 10 [file can-25-5645_figure_s5_suppsf5.pdf]

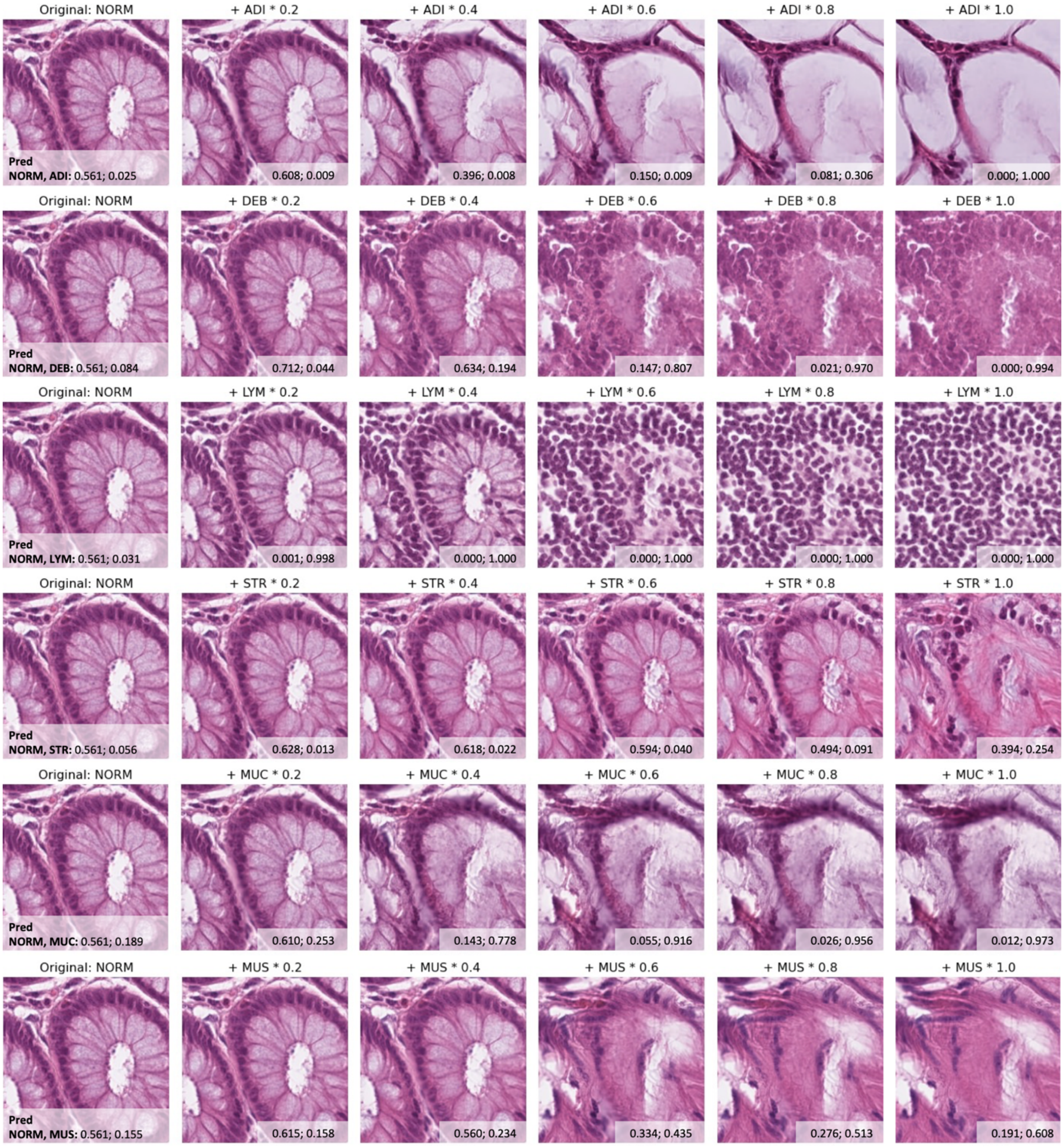


**Figure S5. Examples of counterfactual explanations for the tissue type classifier**, showing how the original histology tile would need to appear to be reassigned to the indicated target class. Each row contains (**left**) the original normal colon mucosa (NORM) tile and shows (**right**) images obtained after progressively larger latent-space shifts toward a target class (manipulation amplitude $\alpha$ sampled at increments of 0.2 from 0.2 to 1.0). The numbers on every tile are the sigmoid scores for two classes. For five classes (adipose [ADI], debris [DEB], lymphocytes [LYM], mucus [MUC], smooth muscle [MUS]), the model's prediction flips to the target when $\alpha \geq 0.6$. In contrast, the cancer-associated stroma (STR) counterfactual images never cross the decision boundary, suggesting that STR tiles lie farther from NORM in latent space, which likely contributes to the model's poorer classification performance on STR (performance metrics shown in Supplementary **Fig. S1B**).
