# Supplementary material for: Counterfactual Diffusion Models Provide Interpretable Explanations of Artificial Intelligence Models in Pathology
Source: Cancer Res. 2026 Jun 17;86(17):4414–33. doi: 10.1158/0008-5472.CAN-25-5645 (PMC13535314; doi:10.1158/0008-5472.CAN-25-5645)
Supplement: Supplementary file 11 [file can-25-5645_figure_s6_suppsf6.pdf]

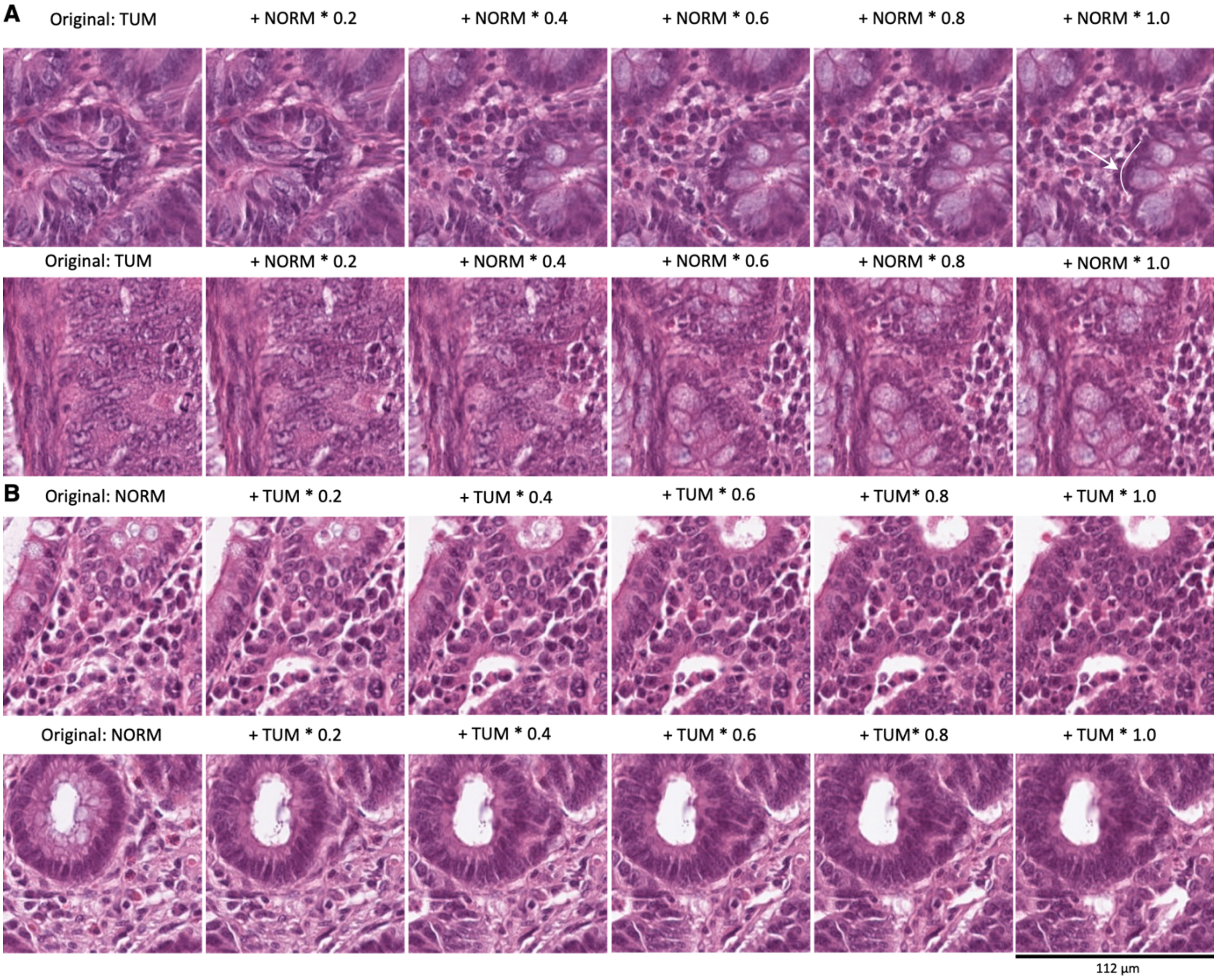

Figure S6. Additional examples of transitions to counterfactual images for the CRC-VAL-HE-7K dataset. The manipulation amplitude (α) is given above each image. (**A**) Dysplastic epithelium tile (TUM) manipulated to healthy colon mucosa (NORM). The model captured the appearance of glands; however, in these examples, fine epithelial-stromal architecture was incompletely restored, with unclear basement membrane delineation and unrealistically smooth boundaries between epithelium and adjacent stroma. (**B**) Normal colon mucosa tile manipulated to dysplastic/tumor epithelium. In the upper synthetic counterfactual image, nuclei showed limited hyperchromasia. Such patterns may reflect the model's internal prioritization of morphological features, for example, the presence of goblet cells might contribute more strongly to class prediction than certain fine-grained structural features or cytologic details.
