# Supplementary material for: Counterfactual Diffusion Models Provide Interpretable Explanations of Artificial Intelligence Models in Pathology
Source: Cancer Res. 2026 Jun 17;86(17):4414–33. doi: 10.1158/0008-5472.CAN-25-5645 (PMC13535314; doi:10.1158/0008-5472.CAN-25-5645)
Supplement: Supplementary file 12 [file can-25-5645_figure_s7_suppsf7.pdf]

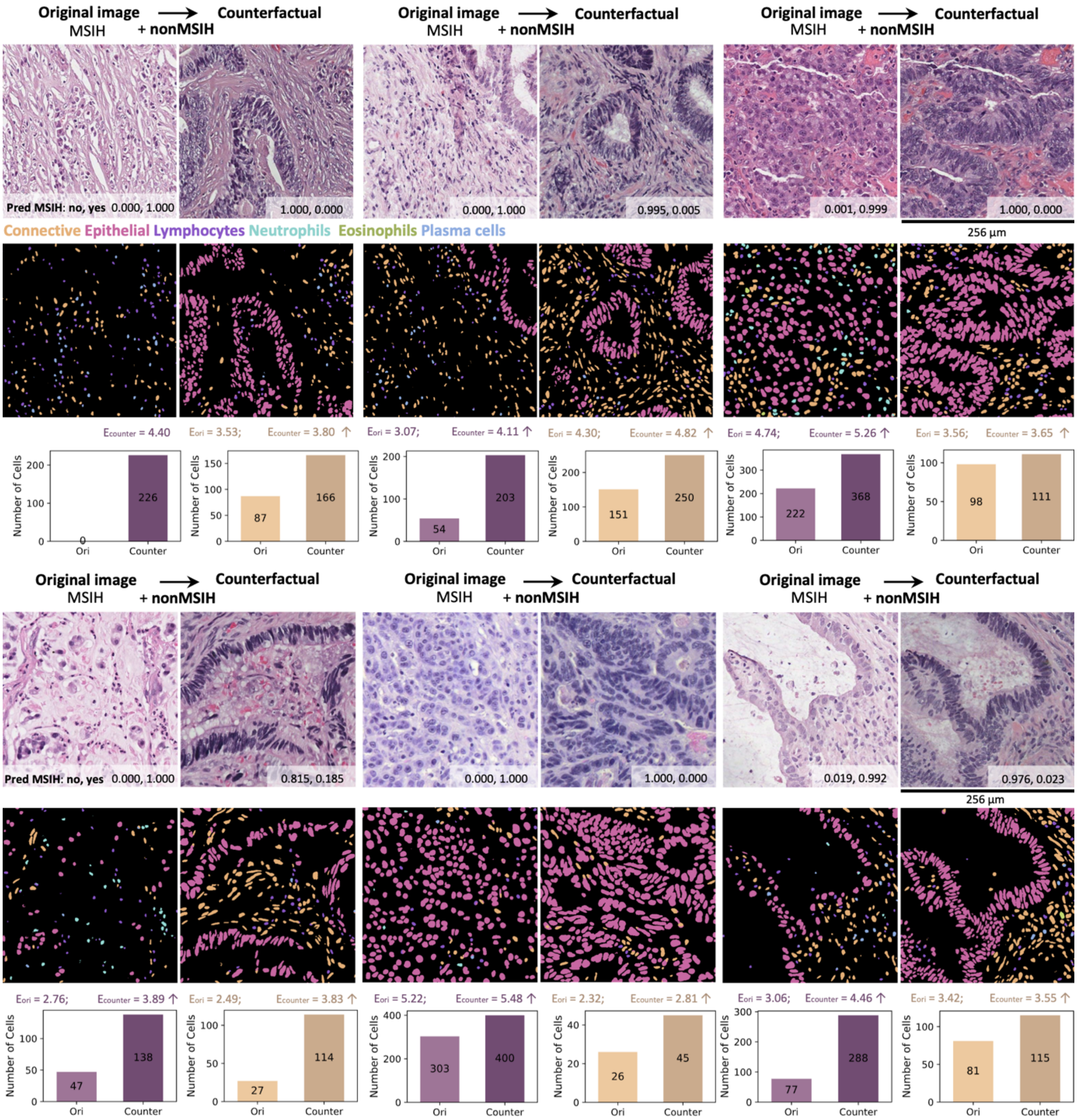

Figure S7. Additional examples of generated counterfactual images for tiles from patients with high microsatellite instability (MSIH). Representative tiles from MSIH patients with corresponding cell segmentation maps showing distinct cell types (connective tissue cells in orange, epithelial cells in pink, lymphocytes in purple, eosinophils in green, and plasma cells in blue) are depicted in two rows. For each case, original MSIH images and their counterfactuals, i.e., images manipulated to be classified as nonMSIH, are compared. Entropy values (*E*) are shown for each image, with arrows indicating an increase (↑) or a decrease (↓) in counterfactual images. The counterfactual images exhibit consistent patterns: an increase in epithelial and connective tissue cell count coupled with higher spatial entropy. Furthermore, a decrease in plasma cells and lymphocytes can be observed qualitatively. Epithelial cells are arranged in a more glandular pattern. The scale bar applies to all images.
