# Supplementary material for: Counterfactual Diffusion Models Provide Interpretable Explanations of Artificial Intelligence Models in Pathology
Source: Cancer Res. 2026 Jun 17;86(17):4414–33. doi: 10.1158/0008-5472.CAN-25-5645 (PMC13535314; doi:10.1158/0008-5472.CAN-25-5645)
Supplement: Supplementary file 13 [file can-25-5645_figure_s8_suppsf8.pdf]

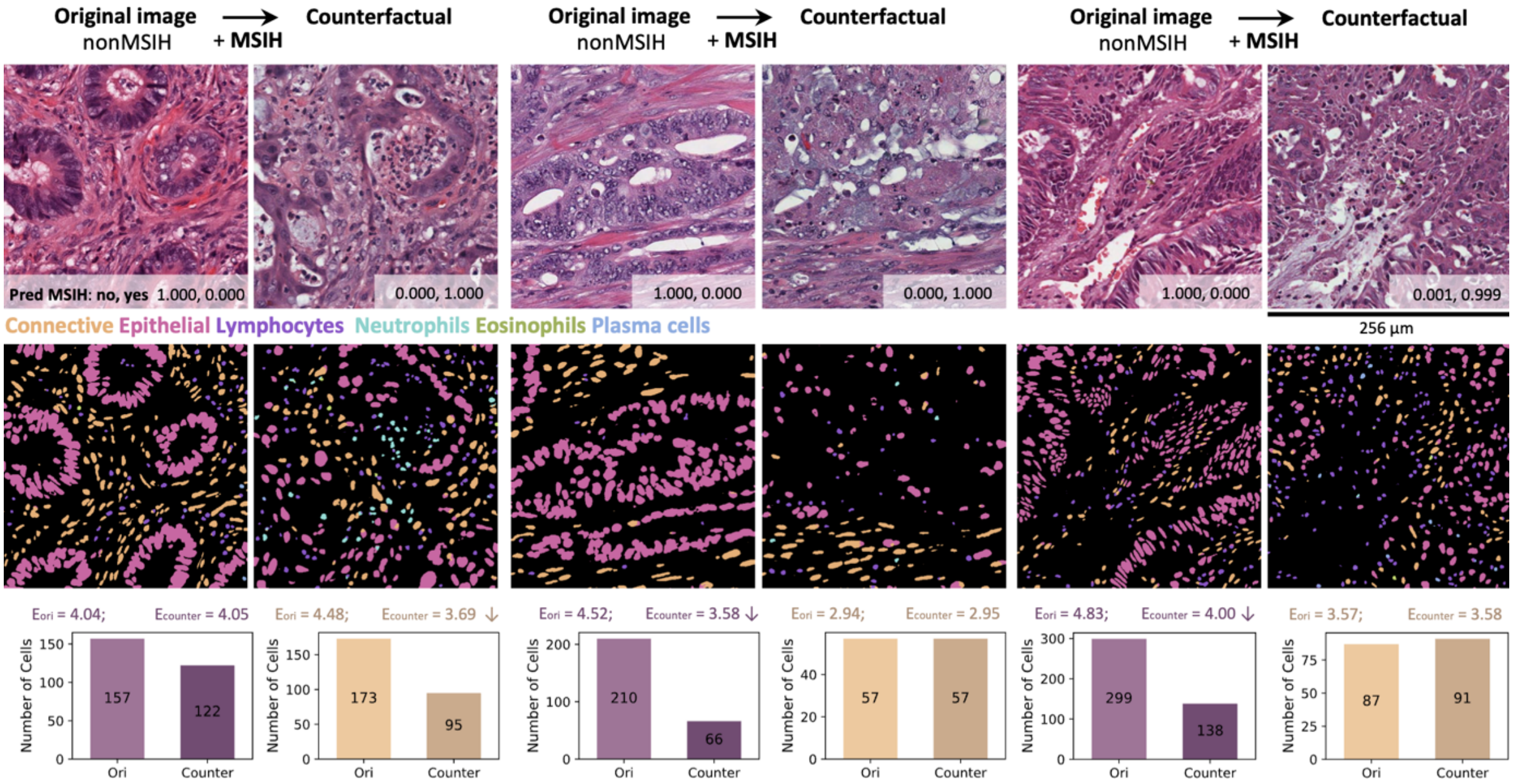

Figure S8. Additional examples of generated counterfactual images for tiles from patients without MSIH status (nonMSIH). The top row contains representative tiles from nonMSIH patients; (**middle**) corresponding cell segmentation maps showing distinct cell types (connective tissue cells in orange, epithelial cells in pink, lymphocytes in purple, eosinophils in green, and plasma cells in blue). For each case, original nonMSIH images and their counterfactuals, i.e., images manipulated to be classified as MSIH, are compared. Entropy values (E) are shown for each image, with arrows indicating an increase (↑) or a decrease (↓) in counterfactual images. In contrast to the examples in Supplementary **Fig. S7**, the counterfactual images show a decrease in epithelial cell count along with lower spatial entropy. Scale bar applies to all images.
