# Supplementary material for: Counterfactual Diffusion Models Provide Interpretable Explanations of Artificial Intelligence Models in Pathology
Source: Cancer Res. 2026 Jun 17;86(17):4414–33. doi: 10.1158/0008-5472.CAN-25-5645 (PMC13535314; doi:10.1158/0008-5472.CAN-25-5645)
Supplement: Supplementary file 14 [file can-25-5645_figure_s9_suppsf9.pdf]

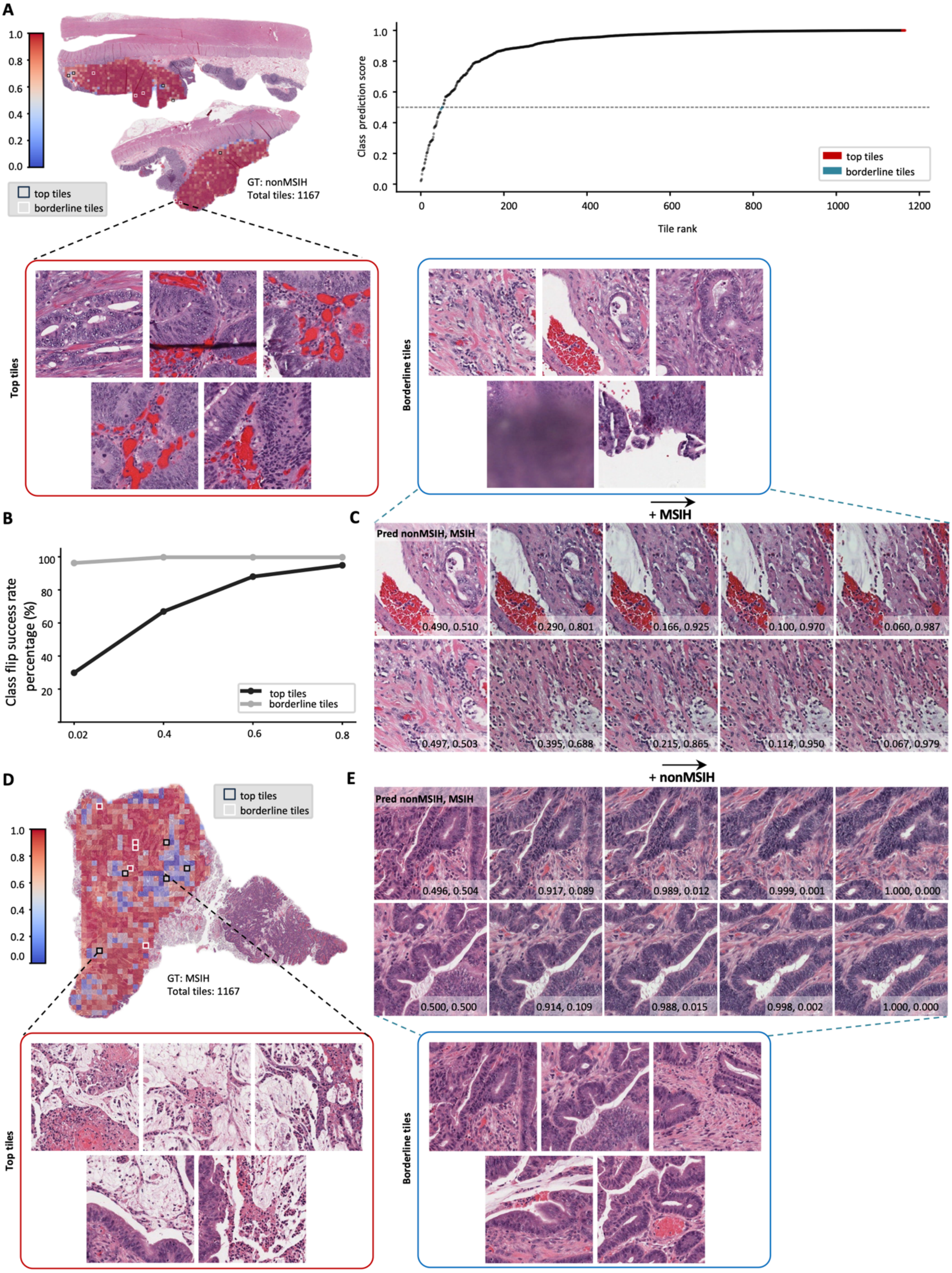

Figure S9. Counterfactual analysis of borderline tiles outside the top-contributing regions in the MSI classifier. (A) Representative nonMSIH WSI with tile-level classifier scores, ranked tile scores, and examples of top-contributing to the prediction and borderline tiles. Borderline tiles were defined as tiles with prediction scores closest to the classifier decision threshold, whereas top tiles corresponded to the highest-

scoring regions for the slide-level prediction. **(B)** Class-flip success rate for generated counterfactual images across increasing manipulation amplitudes for top and borderline tiles. Borderline tiles showed higher flip rates at lower amplitudes, suggesting that these regions are located closer to the classifier decision boundary. **(C)** Representative counterfactual transitions for borderline nonMSIH tiles manipulated toward MSIH. **(D)** Representative MSIH WSI with tile-level classifier scores and examples of selected top and borderline tiles. **(E)** Representative counterfactual trajectories for borderline MSIH tiles manipulated toward nonMSIH.
