# Supplementary material for: Counterfactual Diffusion Models Provide Interpretable Explanations of Artificial Intelligence Models in Pathology
Source: Cancer Res. 2026 Jun 17;86(17):4414–33. doi: 10.1158/0008-5472.CAN-25-5645 (PMC13535314; doi:10.1158/0008-5472.CAN-25-5645)
Supplement: Supplementary file 15 [file can-25-5645_supplementary_tables_suppst1.docx]

## Supplementary Tables

**Table S1. Patient characteristics of the colorectal (TCGA-CRC) cohort.** Patients without available WSIs, with missing spatial resolution value in the metadata (*N* = 26), or missing tumor annotations (*N* = 8) were excluded. WT, wild-type; MUT, mutated.

| **Characteristic** | | **Total *N* patients = 582** | **Train *N* = 474  (479 WSIs)** | **Test *N* = 108  (109 WSIs)** |
| --- | --- | --- | --- | --- |
| **Age (years),  median [IQR]** | 68.0 [58.0, 75.2] | | 68.0 [58.0, 76.0] | 65.0 [57.0, 75.0] |
|  | Missing | 2 | 2 | 1 |
| **Follow-up (days),  median [IQR]** | 555.0 [328.0, 1054.0] | | 617.0 [329.5, 1088.5] | 521.0 [321.5, 925.5] |
|  | Missing | 161 | 160 | 1 |
| **Sex, n (%)** | Male | 302 (51.9%) | 245 (51.7%) | 57 (52.8%) |
|  | Female | 278 (47.8%) | 227 (47.9%) | 51 (47.2%) |
|  | Missing | 2 (0.3%) | 2 (0.4%) | 0 |
| **Organ, n (%)** | Colon | 319 (54.8%) | 233 (49.2%) | 86 (79.6%) |
|  | Rectum | 104 (17.9%) | 83 (17.5%) | 21 (19.4%) |
|  | Missing | 159 (27.3%) | 158 (33.3%) | 1 (0.9%) |
| **Stage, n (%)** | 1 | 69 (11.9%) | 52 (11.0%) | 17 (15.7%) |
|  | 2 | 150 (25.8%) | 106 (22.4%) | 44 (40.7%) |
|  | 3 | 134 (23.0%) | 106 (22.4%) | 28 (25.9%) |
|  | 4 | 57 (9.8%) | 42 (8.9%) | 15 (13.9%) |
|  | Missing | 172 (29.6%) | 168 (35.4%) | 4 (3.7%) |
| **Status, n (%)** | Alive | 335 (57.6%) | 246 (51.9%) | 89 (82.4%) |
|  | Dead | 86 (14.8%) | 68 (14.3%) | 18 (16.7%) |
|  | Missing | 161 (27.7%) | 160 (33.8%) | 1 (0.9%) |
| **Laterality, n (%)** | Left | 225 (38.7%) | 173 (36.5%) | 52 (48.1%) |
|  | Right | 167 (28.7%) | 124 (26.2%) | 43 (39.8%) |
|  | Missing | 190 (32.6%) | 177 (37.3%) | 13 (12.0%) |
| **BRAF, n (%)** | WT | 437 (75.1%) | 339 (71.5%) | 98 (90.7%) |
|  | MUT | 58 (10.0%) | 48 (10.1%) | 10 (9.3%) |
|  | Missing | 87 (14.9%) | 87 (18.4%) | 0 |
| **KRAS, n (%)** | WT | 289 (49.7%) | 235 (49.6%) | 54 (50.0%) |
|  | MUT | 206 (35.4%) | 152 (32.1%) | 54 (50.0%) |
|  | Missing | 87 (14.9%) | 87 (18.4%) | 0 |
| **MSI status, n (%)** | nonMSIH | 360 (61.9%) | 266 (56.1%) | 94 (87.0%) |
|  | MSIH | 61 (10.5%) | 48 (10.1%) | 13 (12.0%) |
|  | Missing | 161 (27.7%) | 160 (33.8%) | 1 (0.9%) |

**Table S2. Distribution of patients in training and testing splits.** Numbers in brackets indicate the number of WSIs, accounting for cases where patients have more than one slide available. During diffusion autoencoder training, the ‘total train’ number of patients was used, whereas during classifier training, patients with unknown/other classes were excluded. LUAD, lung adenocarcinoma; LUSC, lung squamous cell carcinoma; HCC, hepatocellular carcinoma; CCA, cholangiocarcinoma; BRCA, breast invasive carcinoma; ILC, invasive lobular carcinoma; IDC, invasive ductal carcinoma.

|  | **Lung  cancer types** | | **Liver cancer types** | | **TCGA BRCA** | | | |
| --- | --- | --- | --- | --- | --- | --- | --- | --- |
|  | **LUAD** | **LUSC** | **HCC** | **CCA** | **ILC** | **IDC** | **Other** | **Unknown** |
| **Train** | 348 | 350 | 218 | 24 | 108 | 416 | 169 | 128 |
| **Test** | 86 | 87 | 54 | 6 | 23 | 95 | 36 | 71 |
| **Total train** | 698 (775) | | 242 (247) | | 821 (881) | | | |
| **Total test** | 173 (188) | | 60 (61) | | 225 (234) | | | |

**Table S3. Characteristics of the hepatobiliary cohorts used for model development and validation.** FFPE, formalin-fixed and paraffin-embedded; HCC, hepatocellular carcinoma; CCA, cholangiocarcinoma; iCCA, intrahepatic cholangiocarcinoma; pCCA, perihilar cholangiocarcinoma.

| **Characteristic** | | **TCGA Train** | **TCGA Test** | **External Charité** |
| --- | --- | --- | --- | --- |
| **Number of patients** | | 242 | 60 | 496 |
| **Number of WSIs** | | 247 | 61 | 496 |
| **Specimen type** | | FFPE,  resection | FFPE,  resection | FFPE,  resection |
| **Cancer types, n (%)** | HCC | 218 | 54 | 289 (58.3%) |
|  | CCA | 24 | 6 | 207 (41.7%) |
| **CCA subtype, n (%)** | iCCA | 18 (75%) | 6 (100%) | 115 (56.9%) |
|  | pCCA | 3 (12.5%) | 0 | 87 (43.1%) |
|  | Others/ missing | 3 (12.5%) | 0 | 5 |
| **Sex, n (%) HCC / CCA** | Female | 73 (33.5%) / 14 (58.3%) | 13 (24.1%) / 2 (33.3%) | 57 (19.7%) /  75 (36.2%) |
|  | Male | 145 (66.5%) / 10 (41.7%) | 41 (75.9%) / 4 (66.7%) | 220 (76.1%) /  127 (61.4%) |
|  | Missing | 0 | 0 | 12 (4.2%) / 5 (2.4%) |
| **Age (years),  median [IQR]** | HCC | 61.0 (50.0–68.0) | 58.0 (51.0–68.0) | 64.9 (58.4–70.2) |
|  | CCA | 64.0 (54.3–72.3) | 60.5 (57.8–66.3) | 66.0 (56.8–73.2) |
|  | Missing | 3 | 0 | 16 |
| **Grade, n (%)** | HCC | G2: 98 (45.0%)  G3: 88 (40.4%)  G1: 30 (13.8%) | G1: 5 (9.3%)  G2: 27 (50.0%)  G3: 22 (40.7%) | G1: 32 (11.1%)  G2: 174 (60.2%)  G3: 49 (17.0%) |
|  | CCA | G1: 1 (4.2%)  G2: 11 (45.8%)  G3: 11 (45.8%)  G4: 1 (4.2%) | G2: 3 (50.0%)  G3: 3 (50.0%) | G1: 10 (4.8%)  G2: 128 (61.8%)  G3: 52 (25.1%) |
|  | Missing | 2 | 0 | HCC: 34; CCA: 17 |

**Table S4. Statistical characteristics of cell-type percentages across groups: original and counterfactual images of two tissue types (malignant epithelium and normal colon mucosa).** For each cell type, the following metrics are reported: mean (%) ± standard deviation (SD); median (%) and the interquartile range (IQR, %); Hodges-Lehmann (HL) median shift in percentage points with 95% confidence intervals (CI); raw *p* value (*p* raw); corrected *p* value (*p* corr) after Bonferroni correction for multiple comparisons. After correction: *** *p* < 0.001; ** *p* < 0.01; * *p* < 0.05; *ns* – not significant. Qualitative interpretation of Cliff’s delta (δ) to supplement HL shift: |δ| < 0.147 = negligible (*ng*); < 0.33 = small *; < 0.474 = medium **; ≥ 0.474 = large ***. TUM, malignant epithelium; NORM, normal colon mucosa; Manip to TUM, counterfactual images of NORM (synthetic); Manip to NORM, counterfactual images of TUM.

| **Cell type** | **Image type** | **Mean ± SD** | **Median** | **IQR** | **HL shift  (95% CI), pp** | ***p* raw** | ***p* corr** |
| --- | --- | --- | --- | --- | --- | --- | --- |
| **Epithelial** | TUM Original | 94.667 ± 7.192 | 97.186 | 5.744 | -1.57  (-1.83, -1.34)  ** | 1.57 e^-58^ | 9.44 e^-58^  *** |
|  | Manip to TUM | 98.367 ± 2.184 | 99.024 | 1.628 |  |  |  |
|  | NORM Original | 61.911 ± 17.395 | 63.462 | 21.158 | -2.47  (-4.04, -0.82)  *ng* | 3.19 e^-3^ | 1.91 e^-3^ * |
|  | Manip to NORM | 63.940 ± 18.906 | 65.868 | 25.341 |  |  |  |
| **Connective** | TUM Original | 3.705 ± 5.415 | 1.808 | 4.177 | 1.27  (1.07, 1.47)  *** | 5.67 e^-78^ | 3.40 e^-77^  *** |
|  | Manip to TUM | 0.674 ± 1.063 | 0.306 | 0.922 |  |  |  |
|  | NORM Original | 15.606 ± 8.773 | 13.731 | 10.330 | 3.04  (2.38, 3.73)  * | 1.81 e^-19^ | 1.08 e^-18^ *** |
|  | Manip to NORM | 12.211 ± 7.322 | 11.249 | 9.086 |  |  |  |
| **Lymphocytes** | TUM Original | 1.330 ± 2.328 | 0.637 | 1.356 | 0.18  (0.11, 0.24)  * | 1.60 e^-10^ | 9.57 e^-10^  *** |
|  | Manip to TUM | 0.773 ± 1.265 | 0.361 | 0.899 |  |  |  |
|  | NORM Original | 12.963 ± 8.312 | 10.646 | 11.051 | -2.27  (-2.94, -1.51)  * | 9.98 e^-10^ | 5.99 e^-9^ *** |
|  | Manip to NORM | 15.575 ± 9.840 | 13.760 | 10.870 |  |  |  |
| **Plasma cells** | TUM Original | 0.153 ± 0.712 | 0.000 | 0.000 | 0.00  (0.00, 0.00)  *ng* | 4.04 e^-4^ | 2.43 e^-3^  ** |
|  | Manip to TUM | 0.129 ± 0.399 | 0.000 | 0.016 |  |  |  |
|  | NORM Original | 7.853 ± 6.315 | 6.380 | 7.498 | 0.47  (0.00, 0.97)  *ng* | 0.042 | 0.251 *ns* |
|  | Manip to NORM | 7.037 ± 5.380 | 5.989 | 7.454 |  |  |  |
| **Neutrophils** | TUM Original | 0.089 ± 0.458 | 0.000 | 0.000 | 0.00  (0.00, 0.00)  *ng* | 3.45 e^-2^ | 0.207  *ns* |
|  | Manip to TUM | 0.031 ± 0.124 | 0.000 | 0.000 |  |  |  |
|  | NORM Original | 0.045 ± 0.237 | 0.000 | 0.000 | 0.00  (0.00, 0.00)  *ng* | 9.70e^-8^ | 5.82 e^-7^ *** |
|  | Manip to NORM | 0.104 ± 0.375 | 0.000 | 0.000 |  |  |  |
| **Eosinophils** | TUM Original | 0.056 ± 0.321 | 0.000 | 0.000 | 0.00  (0.00, 0.00)  *ng* | 0.582 | 1.00  *ns* |
|  | Manip to TUM | 0.027 ± 0.141 | 0.000 | 0.000 |  |  |  |
|  | NORM Original | 1.622 ± 2.284 | 0.783 | 2.423 | 0.00  (0.00, 0.11)  * | 1.29e^-8^ | 7.73 e^-8^ *** |
|  | Manip to NORM | 1.133 ± 2.174 | 0.094 | 1.581 |  |  |  |

**Table S5. Statistical characteristics of patient-level cell-type percentages across groups: original and counterfactual images of the MSI classifier in colorectal cancer.** For each cell type, we report median (%) and interquartile range (IQR, %); mean difference in percentage points (Δ mean) with 95% bootstrap CI; Cliff’s δ with 95% bootstrap CI; raw *p*-value (two-sided Mann–Whitney U); and Bonferroni-corrected *p* across six cell types within each block. Percentages were computed per tile and averaged within each patient; the table reports patient-level summaries (median [IQR] across patients). Statistical tests and CIs are computed on the per-patient values. **Block 1:** nonMSIH (Real, *N* *=* 94) versus MSIH counterfactuals (CF, *N* = 13). **Block 2:** MSIH (Real, *N* = 13) versus nonMSIH counterfactuals (CF, *N* = 94). CF, counterfactual; MSIH, microsatellite instability-high.

| **Cell Type** | **Median real [IQR]** | **Median CF [IQR]** | **Cliff’s δ  (95% CI)** | **Δ mean (pp) 95% CI** | ***p* raw** | ***p* corr** |
| --- | --- | --- | --- | --- | --- | --- |
| **nonMSIH (N = 94 patients) versus MSIH counterfactuals (N = 13 patients)** | | | | | | |
| **Epithelium** | 63.87 [39.09] | 68.10  [19.59] | 0.155  [-0.108, 0.421] | 9.42  [-0.13, 18.49] | 0.368 | 1.000 |
| **Connective tissue** | 22.88 [28.10] | 18.87  [17.25] | 0.011  [-0.285, 0.316] | -2.09  [-10.16, 6.71] | 0.951 | 1.000 |
| **Plasma cells** | 0.57  [1.63] | 0.29  [0.32] | -0.342  [-0.601, -0.064] | -1.10  [-1.68, -0.52] | 0.047 | 0.281 |
| **Lymphocytes** | 5.89  [9.82] | 4.03  [7.36] | -0.160  [-0.465, 0.152] | -5.82  [-9.81, -1.77] | 0.353 | 1.000 |
| **Eosinophils** | 0.11  [0.43] | 0.11  [0.12] | -0.200  [-0.444, 0.047] | -0.33  [-0.51, -0.18] | 0.245 | 1.000 |
| **Neutrophils** | 0.04  [0.13] | 0.01  [0.03] | -0.334  [-0.604, -0.050] | -0.09  [-0.16, -0.01] | 0.052 | 0.310 |
| **MSIH (N = 13 patients) versus nonMSIH counterfactuals (N = 94 patients)** | | | | | | |
| **Epithelium** | 30.70 [42.95] | 48.25  [39.80] | 0.206  [-0.169, 0.568] | 9.86  [-7.30, 26.73] | 0.231 | 1.000 |
| **Connective tissue** | 17.06 [37.72] | 22.41  [27.21] | -0.113  [-0.476, 0.252] | -5.77  [-20.10, 6.89] | 0.514 | 1.000 |
| **Lymphocytes** | 20.08 [16.34] | 15.83  [15.73] | 0.021  [-0.344, 0.394] | -1.39  [-12.16, 7.73] | 0.905 | 1.000 |
| **Plasma cells** | 3.45  [7.00] | 1.97  [3.13] | -0.092  [-0.499, 0.326] | -1.49  [-4.53, 1.28] | 0.597 | 1.000 |
| **Neutrophils** | 1.75  [2.90] | 0.85  [1.83] | -0.118  [-0.494, 0.273] | -0.97  [-2.66, 0.50] | 0.495 | 1.000 |
| **Eosinophils** | 0.41  [0.94] | 0.28  [0.48] | -0.166  [-0.543, 0.210] | -0.23  [-0.69, 0.14] | 0.336 | 1.000 |

**Table S6. Statistical characteristics of cell-type percentages across groups: original and counterfactual images of hepatobiliary cancer types.** For each cell type, the following metrics are reported: mean (%) ± standard deviation (SD); median (%) and the interquartile range (IQR, %); raw *p*-value; corrected *p*-value with Bonferroni correction for multiple comparisons. HCC, hepatocellular carcinoma; CCA, cholangiocarcinoma; manip to HCC, counterfactual images of CCA (synthetic); manip to CCA, counterfactual images of HCC (synthetic).

| **Cell type** | **Image type** | **Mean ± SD** | **Median** | **IQR** | ***p* raw** | ***p* corr** |
| --- | --- | --- | --- | --- | --- | --- |
| **Epithelial** | HCC Original | 77.928 ± 21.423 | 84.237 | 19.936 | 0.860 | 1.000 |
|  | Manip to HCC | 73.425 ± 28.547 | 84.060 | 32.524 |  |  |
|  | CCA Original | 79.001 ± 14.567 | 81.656 | 17.307 | 0.850 | 1.000 |
|  | Manip to CCA | 78.411 ± 15.555 | 82.740 | 17.623 |  |  |
| **Connective** | HCC Original | 14.279 ± 14.870 | 9.234 | 15.134 | 0.456 | 1.000 |
|  | Manip to HCC | 12.282 ± 11.738 | 8.824 | 17.576 |  |  |
|  | CCA Original | 15.436 ± 10.646 | 12.770 | 14.344 | 0.495 | 1.000 |
|  | Manip to CCA | 14.673 ± 11.698 | 11.067 | 11.980 |  |  |
| **Lymphocytes** | HCC Original | 6.874 ± 10.281 | 3.795 | 6.171 | 0.804 | 1.000 |
|  | Manip to HCC | 12.208 ± 18.863 | 3.049 | 12.428 |  |  |
|  | CCA Original | 5.168 ± 5.016 | 3.615 | 4.237 | 0.254 | 1.000 |
|  | Manip to CCA | 6.323 ± 6.234 | 4.315 | 5.724 |  |  |
| **Plasma cells** | HCC Original | 0.803 ± 2.107 | 0.000 | 0.757 | 0.071 | 0.427 |
|  | Manip to HCC | 1.978 ± 4.634 | 0.220 | 1.982 |  |  |
|  | CCA Original | 0.213 ± 0.534 | 0.000 | 0.124 | 0.609 | 1.000 |
|  | Manip to CCA | 0.300 ± 0.721 | 0.000 | 0.241 |  |  |
| **Neutrophils** | HCC Original | 0.087 ± 0.396 | 0.000 | 0.000 | 0.079 | 0.473 |
|  | Manip to HCC | 0.105 ± 0.206 | 0.000 | 0.090 |  |  |
|  | CCA Original | 0.148 ± 0.298 | 0.000 | 0.153 | 0.599 | 1.000 |
|  | Manip to CCA | 0.213 ± 0.560 | 0.000 | 0.101 |  |  |
| **Eosinophils** | HCC Original | 0.029 ± 0.184 | 0.000 | 0.000 | 0.541 | 1.000 |
|  | Manip to HCC | 0.003 ± 0.016 | 0.000 | 0.000 |  |  |
|  | CCA Original | 0.033 ± 0.109 | 0.000 | 0.000 | 0.362 | 1.000 |
|  | Manip to CCA | 0.080 ± 0.301 | 0.000 | 0.000 |  |  |

**Table S7. Statistical characteristics of cell-type percentages across groups: original and counterfactual images of lung cancer types.** For each cell type, the following metrics are reported: mean (%) ± standard deviation (SD); median (%) and the interquartile range (IQR, %); raw *p*-value; corrected *p*-value with Bonferroni correction for multiple comparisons. * indicates significance at *p* < 0.001 after correction. LUAD, lung adenocarcinoma; LUSC, lung squamous cell carcinoma; manip to LUSC, counterfactual images of LUAD (synthetic); manip to LUAD, counterfactual images of LUSC (synthetic).

| **Cell Type** | **Image type** | **Mean ± SD** | **Median** | **IQR** | ***p* raw** | ***p* corr** |
| --- | --- | --- | --- | --- | --- | --- |
| **Epithelial** | LUAD Original | 73.625 ± 23.669 | 81.255 | 25.964 | $9.646\cdot{10}^{-21}$ | $5.787$ $\cdot{10}^{-20}$ * |
|  | Manip to LUAD | 85.013 ± 16.515 | 91.067 | 16.618 |  |  |
|  | LUSC Original | 67.411 ± 26.451 | 76.779 | 35.111 | 0.154 | 0.922 |
|  | Manip to LUSC | 67.617 ± 30.394 | 79.529 | 34.534 |  |  |
| **Connective tissue** | LUAD Original | 14.737 ± 15.804 | 9.518 | 14.293 | $1.345\cdot{10}^{-24}$ | $8.068$ $\cdot{10}^{-24}$ * |
|  | Manip to LUAD | 7.060 ± 9.201 | 3.661 | 8.578 |  |  |
|  | LUSC Original | 21.770 ± 20.271 | 15.605 | 24.173 | $1.612\cdot{10}^{-3}$ | $9.674\cdot{10}^{-3}$ |
|  | Manip to LUSC | 19.315 ± 20.875 | 11.308 | 23.067 |  |  |
| **Lymphocytes** | LUAD Original | 9.856 ± 11.023 | 6.675 | 8.695 | $3.167\cdot{10}^{-14}$ | $1.900$ $\cdot{10}^{-13}$ * |
|  | Manip to LUAD | 6.241 ± 8.548 | 3.545 | 6.459 |  |  |
|  | LUSC Original | 8.014 ± 9.821 | 4.840 | 7.970 | $3.761\cdot{10}^{-4}$ | $2.256\cdot{10}^{-3}$ |
|  | Manip to LUSC | 10.971 ± 13.553 | 5.997 | 9.555 |  |  |
| **Neutrophils** | LUAD Original | 0.275 ± 1.384 | 0.000 | 0.020 | $7.020\cdot{10}^{-9}$ | $4.212$ $\cdot{10}^{-8}$ * |
|  | Manip to LUAD | 1.289 ± 4.539 | 0.000 | 0.530 |  |  |
|  | LUSC Original | 1.306 ± 4.683 | 0.000 | 0.582 | $2.095\cdot{10}^{-5}$ | $1.257$ $\cdot{10}^{-4}$ * |
|  | Manip to LUSC | 0.228 ± 0.570 | 0.000 | 0.191 |  |  |
| **Plasma cells** | LUAD Original | 1.378 ± 2.569 | 0.441 | 1.410 | $2.061\cdot{10}^{-28}$ | $1.236$ $\cdot{10}^{-27}$ * |
|  | Manip to LUAD | 0.335 ± 0.812 | 0.000 | 0.249 |  |  |
|  | LUSC Original | 1.343 ± 3.078 | 0.142 | 1.381 | $3.529\cdot{10}^{-8}$ | $2.118$ $\cdot{10}^{-7}$ * |
|  | Manip to LUSC | 1.672 ± 2.902 | 0.645 | 2.053 |  |  |
| **Eosinophils** | LUAD Original | 0.128 ± 0.495 | 0.000 | 0.000 | 0.019 | 0.113 |
|  | Manip to LUAD | 0.062 ± 0.239 | 0.000 | 0.000 |  |  |
|  | LUSC Original | 0.155 ± 0.575 | 0.000 | 0.000 | $7.903\cdot{10}^{-3}$ | $4.742\cdot{10}^{-2}$ |
|  | Manip to LUSC | 0.198 ± 0.696 | 0.000 | 0.060 |  |  |

**Table S8. Statistical characteristics of cell-type percentages across groups: original and counterfactual images of breast cancer types.** For each cell type, the following metrics are reported: mean (%) ± standard deviation (SD); median (%) and the interquartile range (IQR, %); raw *p*-value; corrected *p*-value with Bonferroni correction for multiple comparisons. * indicates significance at *p* < 0.001 after correction. IDC, invasive ductal carcinoma; ILC, invasive lobular carcinoma; manip to IDC, counterfactual images of ILC (synthetic); manip to ILC, counterfactual images of IDC (synthetic).

| **Cell Type** | **Image type** | **Mean ± SD** | **Median** | **IQR** | ***p* raw** | ***p* corr** |
| --- | --- | --- | --- | --- | --- | --- |
| **Epithelial** | IDC Original | 63.444 ± 25.710 | 70.512 | 37.118 | 2.061 ⋅ 10^-7^ | 1.237 ⋅ 10^-6^  * |
|  | Manip to IDC | 50.640 ± 24.813 | 52.234 | 32.862 |  |  |
|  | ILC Original | 18.314 ± 20.714 | 9.516 | 36.521 | 0.074 | 0.442 |
|  | Manip to ILC | 20.375 ± 22.808 | 11.969 | 31.717 |  |  |
| **Connective  tissue** | IDC Original | 24.457 ± 17.932 | 20.184 | 20.952 | 3.450 ⋅ 10^-11^ | 2.070 ⋅ 10^-10^  * |
|  | Manip to IDC | 38.991 ± 22.625 | 36.851 | 28.751 |  |  |
|  | ILC Original | 63.207 ± 20.172 | 65.171 | 32.296 | 9.896 ⋅ 10^-9^ | 5.938 ⋅ 10^-8^  * |
|  | Manip to ILC | 50.219 ± 18.883 | 52.167 | 29.512 |  |  |
| **Lymphocytes** | IDC Original | 11.134 ± 15.261 | 5.114 | 11.285 | 3.347 ⋅ 10^-1^ | 1.000 |
|  | Manip to IDC | 9.241 ± 10.294 | 5.981 | 7.063 |  |  |
|  | ILC Original | 14.999 ± 11.210 | 11.481 | 13.775 | 7.488 ⋅ 10^-9^ | 4.493 ⋅ 10^-8^  * |
|  | Manip to ILC | 24.430 ± 17.500 | 17.956 | 22.775 |  |  |
| **Neutrophils** | IDC Original | 0.077 ± 0.281 | 0.000 | 0.042 | 5.169 ⋅ 10^-3^ | 3.102 ⋅ 10^-2^ |
|  | Manip to IDC | 0.184 ± 0.438 | 0.000 | 0.172 |  |  |
|  | ILC Original | 0.034 ± 0.161 | 0.000 | 0.000 | 2.171 ⋅ 10^-7^ | 1.302 ⋅ 10^-6^  * |
|  | Manip to ILC | 0.080 ± 0.273 | 0.000 | 0.050 |  |  |
| **Plasma cells** | IDC Original | 0.837 ± 1.313 | 0.344 | 0.868 | 0.033 ⋅ 10^-1^ | 1.000 |
|  | Manip to IDC | 0.889 ± 1.423 | 0.286 | 0.975 |  |  |
|  | ILC Original | 3.440 ± 4.015 | 2.002 | 4.575 | 7.530 ⋅ 10^-4^ | 4.518 ⋅ 10^-3^ |
|  | Manip to ILC | 4.636 ± 4.713 | 2.991 | 5.251 |  |  |
| **Eosinophils** | IDC Original | 0.052 ± 0.131 | 0.000 | 0.023 | 2.058 ⋅ 10^-1^ | 1.000 |
|  | Manip to IDC | 0.055 ± 0.154 | 0.000 | 0.000 |  |  |
|  | ILC Original | 0.006 ± 0.031 | 0.000 | 0.000 | 1.197 ⋅ 10^-11^ | 7.184 ⋅ 10^-11^  * |
|  | Manip to ILC | 0.260 ± 0.807 | 0.000 | 0.178 |  |  |
